# Selective Gut Microbiota Remodeling Induced by a Traditional Mexican Diet and Exercise Program Improves Metabolic Health and Intestinal Permeability in Adults with Obesity and Affective Symptoms

**DOI:** 10.3390/nu18142308

**Published:** 2026-07-14

**Authors:** María Alejandra Samudio-Cruz, Elizabeth Cabrera-Ruiz, Daniel Cerqueda-García, Pamela D. Rodríguez-Sobrino, Alexandra Luna-Angulo, Carlos Landa-Solís, Samuel Canizales-Quinteros, Jesús Fernando Valencia-León, Blanca López-Contreras, Paul Carrillo-Mora, Ana Luisa Lino-González, Edgar Rangel-López, Marco T. Romero-Sánchez, Yza N. Frías Aguirre, Yessica V. Escobedo-Castro, Silvia H. Pérez Rechy, Rafael Toledo-Pérez, Yaaziel Melgarejo-Ramírez, Laura Sánchez Chapul

**Affiliations:** 1 División de Neurociencias Clínicas, Instituto Nacional de Rehabilitación “Luis Guillermo Ibarra Ibarra”, Mexico City 14389, Mexico; psic.alejandra.samudio@gmail.com (M.A.S.-C.); neuropolaco@yahoo.com.mx (P.C.-M.); ana_onil@yahoo.com.mx (A.L.L.-G.); 2 División de Neurociencias Básicas, Instituto Nacional de Rehabilitación “Luis Guillermo Ibarra Ibarra”, Mexico City 14389, Mexico; elicabreraruiz@gmail.com; 3 Clúster Científico y Tecnológico BioMimic, Red de Manejo Biorracional de Plagas y Vectores, Instituto de Ecología, A.C.—INECOL, Xalapa 91070, Mexico; daniel.cerqueda@inecol.mx; 4 Laboratorio de Enfermedades Neuromusculares, División de Neurociencias Clínicas, Instituto Nacional de Rehabilitación “Luis Guillermo Ibarra Ibarra”, Mexico City 14389, Mexicolunangulo@gmail.com (A.L.-A.); 5 Unidad de Ingeniería de Tejidos, Terapia Celular y Medicina Regenerativa, Instituto Nacional de Rehabilitación “Luis Guillermo Ibarra Ibarra”, Mexico City 14389, Mexico; 293hek@gmail.com; 6 Unidad Periférica de Investigación en Genómica de Poblaciones Aplicada a la Salud, Facultad de Química, Universidad Nacional Autónoma de México (UNAM)/Instituto Nacional de Medicina Genómica (INMEGEN), Mexico City 14610, Mexico; cani@unam.mx (S.C.-Q.); blopez@inmegen.gob.mx (B.L.-C.); 7 Dirección Ejecutiva de Sanidad Naval, Secretaria de Marina, Armada de México, Mexico City 04830, Mexico; jesus_ferval@hotmail.com; 8 Laboratorio de Reprogramación Celular, Instituto Nacional de Neurología y Neurocirugía “Manuel Velasco Suárez”, Mexico City 14269, Mexico; raledg@hotmail.com; 9 Centro de Investigación en Biotecnología Aplicada, Instituto Politécnico Nacional, Santa Inés Tecuexcomac 90700, Mexico; mromeros1900@alumno.ipn.mx; 10 Centro de Estudios Navales en Ciencias de la Salud, Secretaria de Marina, Armada de México, Mexico City 04830, Mexico; drayzagastro@gmail.com; 11 Instituto de Investigación en Ciencias de la Salud, Secretaria de Marina, Armada de México, Mexico City 04830, Mexico; tooparker@yahoo.com (Y.V.E.-C.); labclinsemar@gmail.com (S.H.P.R.); cencis.pr.rtoledo@uninav.edu.mx (R.T.-P.); 12 Laboratorio de Biotecnología, Instituto Nacional de Rehabilitación “Luis Guillermo Ibarra Ibarra”, Mexico City 14389, Mexico; yaazielmr@gmail.com

**Keywords:** obesity, gut microbiota dysbiosis, Traditional Mexican Diet, depression, anxiety

## Abstract

**Background:** Obesity and mental disorders are associated with gut microbiota dysbiosis and gut–brain axis dysfunction. This secondary analysis evaluated the effects of a 12-week weight loss program (WLP) based on a hypocaloric traditional Mexican diet (TMD) and moderate-intensity exercise on gut microbiota, body composition (BC), and metabolic parameters in Mexican adults with obesity with and without depression and anxiety symptoms. **Methods**: A total of 106 adults with obesity were classified based on the presence or absence of depressive and anxiety symptoms into an obesity control group without symptoms (OCG), improved symptoms (OIS), and persistent symptoms (OPS). Stool samples were analyzed by sequencing the V3–V4 region of the 16S rRNA gene. Longitudinal changes in serum biochemistry, BC, intestinal permeability markers, and gut microbiota were analyzed using linear mixed-effects models. Differential taxa were identified using linear discriminant analysis effect size (LEfSe), and associations between bacterial genera, biochemical variables, and dietary components were explored using MaAsLin 2. **Results:** The WLP significantly improved metabolic, inflammatory, intestinal permeability, and BC markers, as well as depressive and anxiety symptoms, although participants remained in WHO grade I obesity. These improvements were accompanied by selective, group-dependent modulation of bacterial genera rather than broad microbiota restructuring. LEfSe identified 22 taxonomic biomarkers: 16 in OPS, 5 in OIS, and 1 in OCG. **Conclusions:** The culturally adapted WLP, which combined a hypocaloric TMD with exercise, promoted improvements in metabolism and BC in all participants, along with group-specific gut microbiota remodeling that may be associated with differential changes in depressive and anxiety symptoms in the context of persistent obesity.

## 1. Introduction

Obesity and mental disorders, specifically depression and anxiety, constitute a global syndemic that severely impairs the quality of life of those affected. It is driven by bidirectional and mutually reinforcing pathophysiological mechanisms [1]. Globally, depressive and anxiety disorders affect approximately one in five adults, while obesity prevalence continues to rise [2]. In Mexico, this public health challenge is particularly important, with a combined prevalence of overweight and obesity reaching 76.2% among adults, according to the national health and nutrition survey ENSANUT 2023 [3], coexisting with a high burden of depressive and anxiety symptoms (ENBIARE 2021) [4,5]. Longitudinal evidence supports the bidirectional association between these conditions: depression increases the risk of developing obesity by approximately 37%, while obesity raises the risk of subsequent depression by about 40% [6,7] and is consistently associated with a higher likelihood of anxiety disorders [7,8]. This epidemiological convergence is explained by shared biological mechanisms, including chronic low-grade inflammation, dysregulation of the hypothalamic–pituitary–adrenal (HPA) axis, structural and functional alterations in brain regions involved in reward processing and mood regulation, and significant shifts in the gut microbiota [6,9].

Emerging evidence identifies the gut–brain axis as a key biological interface linking metabolic and mental health [10,11,12,13,14], with conditions that are significantly influenced by modern lifestyle behaviors, including sedentary habits and the consumption of ultra-processed calorie-dense diets [4,15,16,17,18,19]. Physical inactivity not only increases adiposity but also impairs neurobiological pathways, reducing neurotrophic factors and altering neurotransmitter signaling [16]. Similarly, diets high in saturated fats and refined sugars trigger low-grade systemic inflammation, disrupt HPA axis function, and alter the production of neuroactive metabolites. These processes induce metabolic dysregulation and adverse shifts in gut microbiota composition, directly contributing to the pathophysiology of depression and anxiety [4,10,17,20,21].

Conversely, structured lifestyle interventions incorporating a balanced diet and regular exercise promote weight loss, restore metabolic health, alleviate symptoms of depression and anxiety, and positively influence gut microbiota [22]. In this sense, while the Mediterranean diet is widely recognized for its high fiber and omega-3 fatty acid content [23], the Traditional Mexican Diet (TMD) has emerged as a robust alternative [24]. The TMD, characterized by staples such as maize, beans, nopal, squash, chili, and avocado, is rich in dietary fiber and bioactive compounds associated with improved mental health outcomes [18,25,26,27,28]. These benefits are mediated, in part, by the modulation of the gut microbiota, leading to increased microbial diversity and a favorable shift towards anti-inflammatory taxa [10,18,29]. Additionally, regular aerobic, resistance, or combined exercise provides a synergistic effect in reducing depressive and anxiety symptoms, with evidence supporting a dose–response relationship up to an optimal range of physical activity [17,30,31].

We recently characterized gut microbiota signatures in Mexican adults with obesity and comorbid depressive or anxiety symptoms, identifying 30 taxonomic biomarkers with clinical potential for therapeutic modulation [32]. Despite the severity of this syndemic in Mexico, there is a notable scarcity of clinical trials evaluating culturally adapted nutritional interventions [33]. While the TMD is recognized for its prebiotic potential and high concentrations of bioactive compounds [4,18,24,25], its effect on the gut–brain-metabolic axis when combined with physical activity has yet to be fully elucidated. The aim of the present study was to conduct a secondary analysis of a recently published study [32] to evaluate the impact of a structured 12-week weight loss program (WLP) based on a hypocaloric TMD and moderate-intensity exercise on gut microbiota, body composition (BC), and metabolic markers in Mexican adults with obesity with and without depressive and anxiety symptoms.

## 2. Materials and Methods

### 2.1. Participants

One hundred and forty-seven individuals of both sexes (33 women and 114 men) with diagnosed obesity were recruited from the Mexican Navy. These individuals participated in a structured 12-week WLP organized by the Mexican Navy. The inclusion criteria were body mass index (BMI) > 30 kg/m^2^, age between 18 and 60 years, controlled comorbidities or none, and low cardiometabolic risk or none. Participants with diarrhea, who did not adhere to the diet or exercise program, who had musculoskeletal injuries that prevented them from continuing the exercise program, who chose to discontinue the study, or who had taken antibiotics 3 months before enrollment were excluded. The study was approved by the Research and Ethics Committee of the National Institute of Rehabilitation “Luis Guillermo Ibarra” (CONBIO-ETICA-09-CEI-03120171207). All Navy personnel were informed of the benefits and risks of the study before signing an institutional informed consent form to enable them to participate in the study.

### 2.2. Structured Weight Loss Program

Participants completed a 12-week structured WLP that combined a hypocaloric TMD with moderate-intensity exercise. All participants stayed in the same location for the 12-week program. The dietary intervention was based on an assessment of each participant’s habitual food intake, including the foods most frequently consumed 3–4 days per week and the macronutrient distribution of their diet. To promote healthy weight loss, a calorie-restricted diet (1300–1400 kcal/day) consisting only of traditional Mexican foods was provided [24], with a macronutrient distribution of 50% carbohydrate, 30% fat, 20% protein, and a fiber intake of approximately 25 g/day. The baseline and final macro- and micronutrient intakes were calculated daily using the Mexican Equivalent Food System (SMAE) [34]; they are presented in Table A1. Exercise intensity was individually established according to the clinical guidelines for adult exercise to 50–60% [35] of each participant’s heart rate reserve (3.0–5.9 METs) [36], considering the baseline clinical profiles, including age, comorbidities, and physical fitness criteria [35]. Adherence was strictly monitored, with a minimum threshold of 80% required for inclusion in the study.

### 2.3. Assessment of Depressive and Anxious Symptoms

To assess the presence and severity of psychological symptoms, a psychologist from the Mexican Navy administered the Beck Depression Inventory (BDI) and the Beck Anxiety Inventory (BAI), individually in separate cubicles, at the beginning of the WLP and again 12 weeks later [32]. Both instruments consist of 21 self-report items designed to evaluate typical attitudes and manifestations of the respective disorders, with higher scores indicating greater symptom severity.

### 2.4. Physical Fitness Criteria

The stratification of cardiovascular risk was determined before starting the WLP by clinical assessment, including spirometry and resting electrocardiogram. Mexican Navy medical personnel performed these evaluations.

Body composition (BC) was determined at the beginning and end of the WLP by bioelectrical impedance using a SECA Medical Body Composition Analyzer, in addition to waist circumference measurement. Weight, height, and circumference were determined in accordance with the guidelines of the International Society for the Advancement of Kinanthropometry (ISAK). To avoid technical measurement errors, all measurements were taken on the same day, in the same session, and under the same conditions. Before the anthropometric measurements, participants were asked not to exercise, to fast for 12 h, to wear only shorts without shoes, and to not apply any lotions, oils, or body creams. Measurements were performed by two ISAK Level 2 certified anthropometricians from the Mexican Navy.

Body Mass Index was calculated by dividing weight (in kg) by height (in square meters) (kg/m^2^). Height and weight were measured using a portable stadiometer (Seca model 213, Hamburg, Germany) and a SECA Medical Body Composition Analyzer, respectively.

### 2.5. Laboratory Data Collection

Blood samples were obtained by venipuncture after a 12-h fasting period (Vacutainer, Becton, Dickinson and Company, Franklin Lakes, NJ, USA) at the beginning and at the end of the WLP. The serum was immediately separated by centrifugation and stored at −80 °C until use. Serum biochemistry and liver parameters were assessed at the Naval Medical Center Clinical Pathology Laboratory.

### 2.6. Neurotrophic and Intestinal Permeability Biomarkers

Brain-derived neurotrophic factor (BDNF) and zonulin concentrations in serum were quantified using a commercial ELISA kit (Human BDNF ELISA Kit, Cat. No. EH42RB and Human zonulin ELISA Kit, Cat. No. EEL072, Thermo Fisher Scientific Inc., Waltham, MA, USA) according to the manufacturer’s protocol. BDNF immunoassays had an intra- and inter-assay coefficient of variance <10% and <12%, respectively, and an analytical sensitivity of 80 pg/mL. The zonulin immunoassay had an intra- and inter-assay coefficient of variance <10% and an analytical sensitivity of 0.47 ng/mL.

### 2.7. Stool Sample Collection and DNA Extraction

Participants were given a kit containing a sterile, dry plastic container and a scoop. The instructions were to collect the first stool of the morning, to place a small portion of the sample in the container, avoiding contamination with urine or toilet water, to tightly close and clearly label the container. The samples were kept refrigerated and transported to the laboratory on ice. Upon arrival, four aliquots of 180–200 mg were prepared and stored at −80 °C until processing. Total bacterial DNA was extracted from 200 mg of feces using the QIAamp DNA Stool Mini Kit (Qiagen, Hilden, Germany) according to the manufacturer’s protocol with a final elution volume of 100 µL. The eluates were stored at −20 °C until further analysis. DNA concentration and purity were determined using a Qubit fluorometer (Thermo Fisher Scientific, Waltham, MA, USA) and a Nano Drop One spectrophotometer (Thermo Fisher Scientific, Waltham, MA, USA), respectively.

### 2.8. Gut Microbiota Analysis

Gut microbiota composition was analyzed by sequencing the V3–V4 hypervariable region of the 16S rRNA gene using Illumina MiSeq paired-end technology (2 × 300 bp) as previously reported [32]. Amplicons were generated with primers 341F/805R, purified, indexed with Nextera XT adapters, and quantified using Qubit fluorometry. Equimolar pooled libraries (4 pM) were sequenced on the Illumina MiSeq 500 platform after standard PCR amplification and quality verification procedures [32].

### 2.9. Bioinformatic and Statistical Analysis

The paired-end raw sequences in fastq format were processed using the QIIME2 pipeline (v.2023.9) [37]. Amplicon sequence variants (ASVs) were denoised and trimmed using the DADA2 plugin [38]. The taxonomic classification of Amplicon sequence variants (ASVs) was performed with the classify-consensus-v-search plugin [39], using the SILVA v138 database [40]. A phylogenetic tree of representative ASVs was constructed to calculate the UniFrac distance matrix using the plugin align-to-tree-mafft fasttree, which aligns sequences using MAFFT (v.7.520) [41] and constructs a tree using FastTree2 (v. 2.2.0) [42]. The resulting ASV frequency table and phylogeny were exported to the R environment (v.4.1.2) for further analysis. In R, the phyloseq package [43] was used to rarefy the samples to a sequencing depth of 14,000 counts. We calculated the alpha diversity indices, Shannon and observed species, and significant differences were tested using an independent-samples *t*-test. The UniFrac distance matrix was used to calculate beta-diversity metrics, which were visualized in a PCoA (principal coordinates analysis). A PERMANOVA analysis was used to test for differences in beta diversity between groups using the Vegan library [44]. A linear discriminant effect size (LEfSe) analysis was performed to identify taxa with different abundances in each group, using an LDA effect cut-off of >2 and a *p*-value < 0.05. Association analyses between bacterial genera and clinical and nutritional variables were performed using the MaAsLin2 package (v.3.21) [45]. For each biochemical and nutritional variable and its relative abundance, only the linear model was fitted separately, using total-sum scaling normalization, log transformation, and the Benjamini–Hochberg correction. Associations with q < 0.05 were considered significant.

### 2.10. Statistical Analysis

Continuous variables are expressed as mean ± standard deviation (SD), while categorical data are presented as frequencies and percentages. Wilcoxon signed-rank tests were performed to evaluate pre- and post-intervention changes in the median relative abundance (MRA) of taxa (phylum, class, and genus) separately for the OCG, OIS, and OPS groups. Statistical significance was set at *p* < 0.05. Longitudinal changes in serum biochemistry, liver function markers, BC, intestinal permeability, neurotrophic factor and gut microbiota remodeling were analyzed using Linear Mixed-Effects Models (LMMs), which are robust to unbalanced designs and unequal sample sizes across groups while accounting for within-subject correlations in repeated measurements. In addition, LMMs allow inclusion of all available observations without excluding participants with partially missing data, thereby improving statistical power and reducing bias compared with traditional repeated-measures ANOVA. For each outcome, group (OCG, OIS, OPS), time (before vs. after intervention), and their interaction (group-by-time) were included as fixed effects, with a random intercept for subjects to account for within-subject correlation. Age and sex were included as covariates in all models to adjust for potential confounding effects. Post hoc pairwise comparisons of estimated marginal means were conducted with Bonferroni adjustment. To control for multiple testing across biomarkers, false discovery rate (FDR) correction using the Benjamini–Hochberg procedure was applied to interaction *p*-values. Analyses were performed using IBM SPSS Statistics for Windows, version 21 (IBM Corp., Armonk, NY, USA).

## 3. Results

The study included a total of 106 individuals from the Mexican Navy (83 males (78.3%) and 23 females (21.7%)) with a mean age of 39.44 years (±7.32) and diagnosed with obesity (BMI ≥ 30 kg/m^2^). Before the WLP, 34% of participants exhibited depressive and/or anxiety symptoms; at the end of the WLP, 22% showed improvement in these symptoms.

The characteristics of the study population are shown in Table 1. According to the cut-off points of the BDI (10 points) and the BAI (6 points), participants were divided into three groups based on the presence or absence of depressive and anxiety symptoms: a control group of obese participants without symptoms of depression or anxiety before and after WLP (OCG), a group of obese participants who showed improvement in depressive or anxiety symptoms after WLP (OIS), and a group of obese participants with persistent depressive or anxiety symptoms after WLP (OPS).

OCG is the control group of obese participants without symptoms of depression or anxiety before and after the WLP. OIS is the group of obese participants who showed improvement in depressive or anxiety symptoms after the WLP. OPS is the group of obese participants with persistent depressive or anxiety symptoms after the WLP.

### 3.1. Effects of the Intervention on Metabolic, Inflammatory, Gut Barrier Integrity, Neurotrophic Biomarkers, and BC

The results of the longitudinal changes in BC, serum biochemistry, liver function markers, gut barrier integrity, and neurotrophic biomarkers are presented in Table 2. Significant main effects of time were identified for BC and key metabolic, inflammatory, and gut barrier integrity biomarkers. The group-by-time interaction showed differential responses, though not statistically significant, indicating that all beneficial effects were broadly similar across the three groups. For BC outcomes, a significant main effect of time was observed for body weight, BMI, body fat %, fat-free mass %, and muscle mass. Body weight, BMI, body fat %, and muscle mass significantly decreased after the intervention, while fat-free mass % increased (all *p* < 0.001). No significant main effect of group or group-by-time interactions was detected, indicating that weight loss and BC changes were comparable across the three groups.

For serum biochemistry, VLDL-cholesterol, total cholesterol, and triglycerides decreased significantly following the intervention (all *p* < 0.001), independently of group allocation. No significant group-by-time interactions were observed, indicating a similar lipid-lowering effect across the OCG, OIS, and OPS groups. Fasting insulin showed a marked reduction over time (*p* < 0.001), suggesting an overall improvement in insulin sensitivity. Similarly, C-reactive protein (CRP) decreased significantly (*p* = 0.006), supporting a reduction in systemic inflammation following the intervention. No significant changes were observed for fasting glucose, HDL-cholesterol, LDL-cholesterol, creatinine, or cortisol. Blood urea nitrogen and uric acid decreased significantly (*p* < 0.001 and *p* = 0.034, respectively).

For hepatic enzymes, a significant main effect of time was observed, with reductions in AST (Aspartate aminotransferase), ALT (Alanine aminotransaminase), and GGT (Gamma-Glutamyl Transferase) (all *p* < 0.001), as well as ALP (alkaline phosphatase) (*p* = 0.003), reflecting a consistent decrease in liver enzyme levels after the intervention. A significant main effect of the group was observed only for total bilirubin, indirect bilirubin, and albumin (*p* < 0.05). As these variables did not show significant group-by-time interactions, the observed differences were interpreted as baseline intergroup variability rather than effects attributable to the intervention.

Another important finding was the significant reduction in serum zonulin over time, suggesting improved intestinal permeability in all groups following the intervention.

### 3.2. Gut Microbiota Structure

#### 3.2.1. Alpha and Beta Diversity

**Figure A1:**
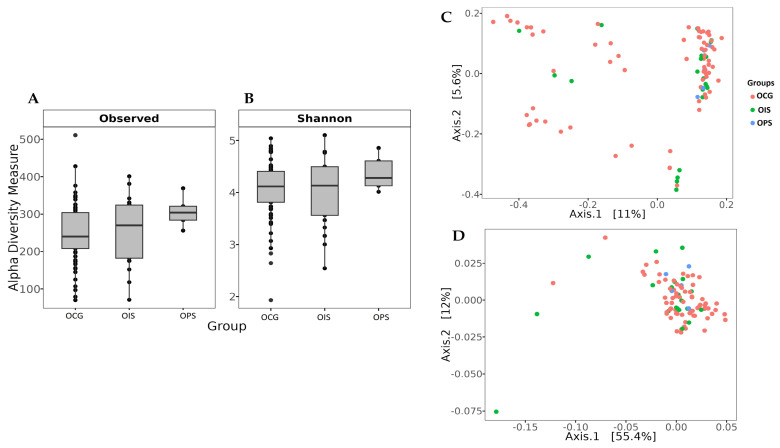
Alpha and beta diversity in fecal samples of all groups after the WLP. (**A**) Box plot showing the alpha diversity of the bacterial communities based on observed OTUs. (**B**) The Shannon diversity index between the groups. (**C**) PCoA plot of unweighted UniFrac distances and (**D**) weighted UniFrac distances of the groups using the rarefied OTU matrix. Orange dots represent the control group (OCG), green dots represent the group who improved depressive and anxiety symptoms (OIS), and blue dots represent the group with persistent depressive and/or anxiety symptoms (OPS).

The gut microbiota richness and diversity for each group after the WLP are shown in Figure A1. Both the observed operational taxonomic units (OTUs) and the Shannon index (Figure A1A,B), as well as the PERMANOVA results from the distance matrices (Figure A1C,D), indicate that there were no significant differences in alpha and beta diversity among the groups. Specifically, the Shannon index did not differ significantly between the OCG with improved symptoms (OIS) and the group with persistent symptoms (OPS). Similarly, the PCoA based on both unweighted and weighted UniFrac distances (Figure A1C,D) showed no distinct clustering among the groups.

#### 3.2.2. Effects of the Weight Loss Program on the Overall Composition of the Gut Microbiota

**Figure 1:**
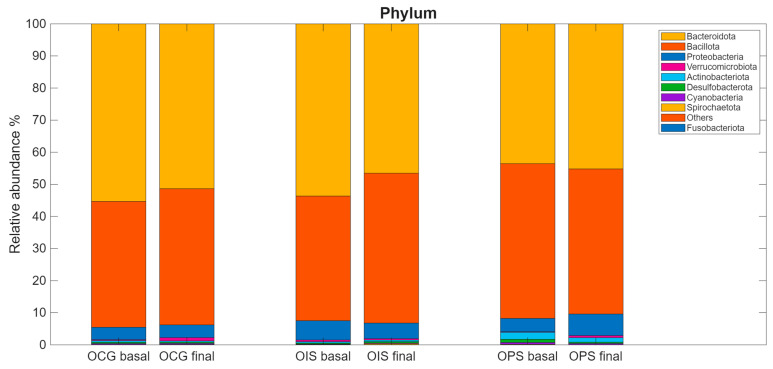
Relative abundance of bacterial phyla in fecal samples before and after the weight loss program. The stacked bar chart shows the distribution of each bacterial phylum in each group. The colored bars within the legend and the stacked bars are ordered from highest to lowest relative abundance.

**Figure 2:**
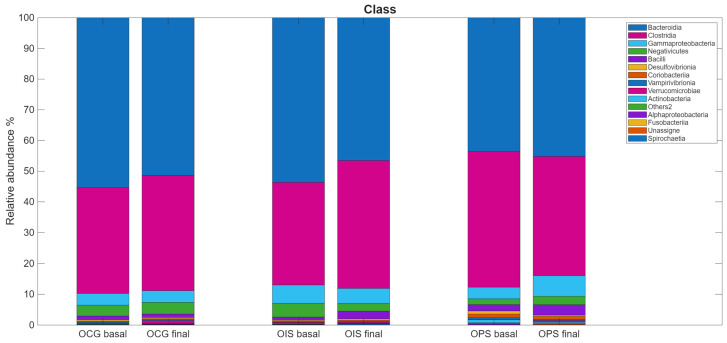
Relative abundance of bacterial classes in fecal samples before and after the weight loss program. The stacked bar chart shows the distribution of each bacterial class in each group. The colored bars within the legend and the stacked bars are ordered from highest to lowest relative abundance.

**Figure 3:**
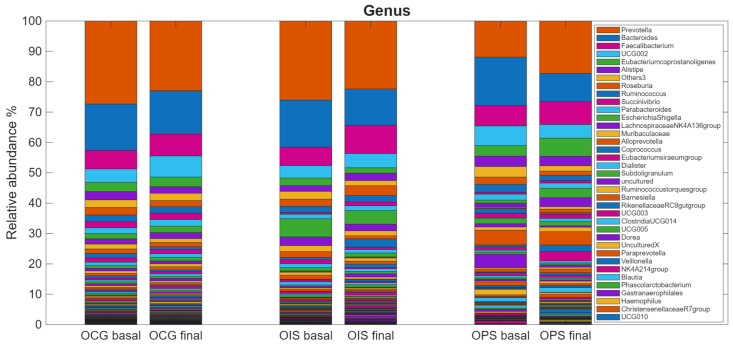
Relative abundance of bacterial genera in fecal samples before and after the weight loss program. The stacked bar chart shows the distribution of each bacterial genus in each group. The colored bars within the legend and the stacked bars are ordered from highest to lowest relative abundance.

The B/B ratio and MRA at the phylum, class, and genus levels for each group, before and after WLP, are shown in Table A2 and Figure 1, Figure 2 and Figure 3, respectively. At the phylum level, the gut microbiota in all groups was dominated by *Bacteroidota* and *Bacillota*, which remained relatively stable throughout the intervention. Other phyla, including *Proteobacteria*, *Actinobacteriota*, *Desulfobacterota*, *Cyanobacteria*, and *Fusobacteriota*, also showed minimal variation. A modest increase in *Verrucomicrobiota* was observed in the OCG. At the class level, *Bacteroidia* and *Clostridia* remained as the predominant taxa across all groups, with only minor fluctuations in less abundant classes. More pronounced changes were detected at the genus level. In OCG, post-intervention shifts included increases in UCG002, *Escherichia*–*Shigella*, *Barnesiella*, *Dorea*, *Paraprevotella*, *Gastranaerophilales*, and *Fusobacterium*, along with reductions in *Roseburia*, *Ruminococcus*, *Coprococcus*, *Streptococcus*, *Klebsiella*, and *Oscillibacter*. In OIS, the intervention was characterized by increases in the *Rikenellaceae* RC9 gut group, UCG005, *Veillonella*, and *Haemophilus*, together with decreases in *Barnesiella*, UCG003, and *Weissella*. In contrast, the OPS group exhibited a comparatively stable genus-level profile (Table A2).

The results of the LMM evaluating longitudinal shifts in gut microbiota composition are presented in Table 3. The LMM adjusted the pre-WLP mean reported in Table A2 by separating the overall population signal from the specific variability within each subject or group and accounting for the covariates sex and age. At the phylum level, no significant main effects of time or group-by-time interactions were observed for the *Bacillota*/*Bacteroidota* ratio or the dominant phyla (*Bacteroidota*, *Bacillota*, *Proteobacteria*, and *Verrucomicrobiota*; all *p* > 0.05). However, significant main group effects were detected for *Actinobacteriota* (*p* = 0.005) and *Desulfobacterota* (*p* = 0.030), indicating baseline differences among groups.

At the class level, a significant main effect of time was observed for *Bacilli* (*p* = 0.048) along with a significant group-by-time interaction (*p* = 0.008), suggesting a differential response to the intervention across groups. A similar interaction was noted for *Fusobacteriia* (*p* = 0.018). Additionally, significant group effects were also identified for *Coriobacteriia* (*p* = 0.013), *Alphaproteobacteria* (*p* = 0.046), *Desulfovibrionia* (*p* = 0.030), and *Actinobacteria* (*p* = 0.005).

At the genus level, most dominant taxa—including *Prevotella*, *Bacteroides*, *Faecalibacterium*, *Roseburia*, and *Ruminococcus*—remained stable, showing no significant longitudinal changes. Significant group effects were observed for *Escherichia-Shigella* (*p* = 0.004), *Barnesiella* (*p* = 0.02), *Dorea* (*p* < 0.001), *Clostridium sensu stricto* (*p* = 0.003), *Lachnospiraceae* UCG001 (0.050), *Lactobacillus* (*p* = 0.005), and *Raoultella* (*p* = 0.012), indicating baseline differences between cohorts. In contrast, significant time and/or interaction effects were detected for *Dorea* (time: *p* = 0.004; interaction: *p* < 0.001), UCG003 (time: *p* = 0.016; interaction: *p* = 0.044), *Streptococcus* (time: *p* = 0.002; interaction: *p* = 0.001), *Bilophila* (group: *p* = 0.005; time: *p* = 0.016; interaction: *p* = 0.037), *Oscillibacter* (time and interaction: *p* < 0.001), *Megamonas* (time: *p* = 0.001; interaction: *p* = 0.005), *Bifidobacterium* (group: *p* = 0.008; interaction: *p* = 0.020), *Rikenellacea* (time: *p* = 0.030; interaction: *p* = 0.011), and *Acidaminococcus* (time: *p* = 0.035; interaction: *p* = 0.023). Additional interaction effects were observed for *Lachnospiraceae* UCG001 (*p* = 0.042) and *Anaerovibrio* (*p* = 0.026).

Overall, the intervention did not induce broad restructuring of the dominant phyla or the *Bacillota*/*Bacteroidota* ratio. Instead, it was associated with selective, group-dependent modulations of specific bacterial genera, suggesting a taxon-specific rather than a global shift in gut microbiota composition following the weight loss program.

#### 3.2.3. Associations Between Taxonomic Profile and Measured Variables

**Figure 4:**
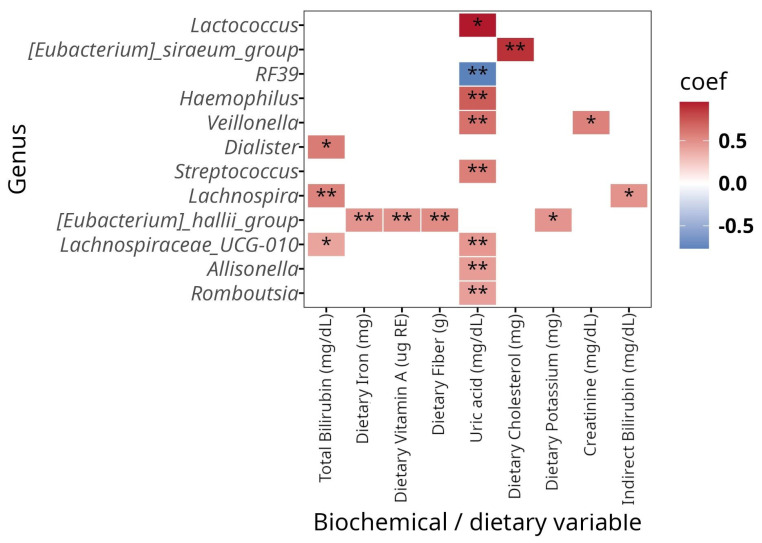
Heatmap illustrating significant associations between the relative abundance of several genera with serum biochemical parameters and dietary components (* *p* < 0.05 and ** *p* < 0.01). Color and +/− symbols indicate the direction and magnitude of the adjusted effects.

Associations between ASV abundance, serum biochemistry, BC, and dietary micronutrients were assessed using linear mixed-effects models and the MaAsLin2 package (Figure 4). The heatmap reveals selective, statistically significant relationships between specific gut microbial genera and host biochemical and dietary variables, supporting a functionally structured microbiota–host interaction. There was a notable pattern of positive associations (red coefficients) between ASVs belonging to *Lactococcus*, *Haemophilus*, *Veillonella*, *Streptococcus*, *Lachnospiraceae* UCG-010, *Allisonella*, and *Romboutsia* with uric acid, whereas only one negative association (blue coefficient) was observed for RF39. These findings suggest a coordinated microbial signature linked to purine metabolism and systemic metabolic status, with potential implications for cardiometabolic risk.

For hepatic markers, total bilirubin showed positive associations with ASVs belonging to *Dialister*, *Lachnospira*, and *Lachnospiraceae* UCG-010, while indirect bilirubin was specifically associated with *Lachnospira*. These relationships suggest a potential role for these taxa in enterohepatic metabolism and liver–microbiota crosstalk.

Additionally, *Veillonella* showed a positive association with creatinine, suggesting a possible link with host energy metabolism or renal function. While this may reflect adaptive or regulatory microbial responses, it could also indicate context-dependent contributions to oxidative stress and metabolic homeostasis.

Notably, the *Eubacterium hallii* group showed consistent positive associations with fiber intake and micronutrients, including dietary potassium, iron, and vitamin A, reinforcing its recognized role as a SCFA-producing taxon responsive to dietary quality. Similarly, the *Eubacterium siraeum* group was positively associated with dietary cholesterol.

#### 3.2.4. The Weight Loss Program Led to Enriched Taxa That Define Each Group

**Figure 5:**
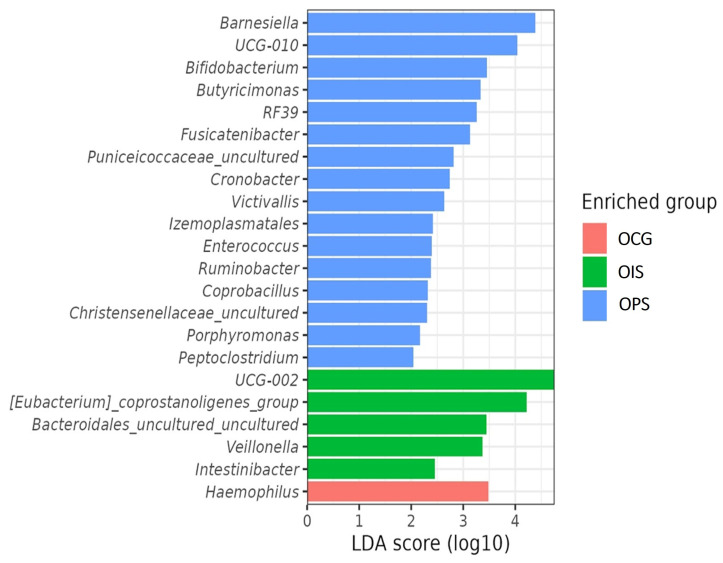
Linear discriminant effect size analysis describing the characteristic ASVs of the taxonomic profile of the control group (OCG), individuals who improved depressive and/or anxious symptoms (OIS), and individuals with persistent depressive and/or anxious symptoms (OPS). Only ASVs with LDA scores (log10) > 2 are shown.

The LEfSe analysis identified 22 discriminant taxonomic biomarkers in our population after the WLP, indicating distinct microbial signatures for each group (Figure 5). The OPS group had the highest number of enriched taxa (16) belonging to *Bacteroidota*, *Bacillota*, *Actinobacteriota*, *Proteobacteria*, and *Verrucomicrobiota* phyla, and the greatest overall LDA scores. The ASVs with the highest LDA scores and greatest discriminative power belong to the genera *Barnesiella*, *Lachnospiraceae* UCG-10, *Bifidobacterium*, *Butyricimonas*, and RF39, an unclassified *Bacillales*. Additional taxa enriched in the OPS group included *Fusicatenibacter*, *Cronobacter*, *Victivallis*, *Izemoplasmatales*, *Enterococcus*, *Ruminobacter*, *Coprobacillus*, *Christensenellaceae*_uncultered, *Porphyromonas*, and *Peptoclostridium*. Notably, several of these genera are recognized producers of short-chain fatty acids (SCFAs), suggesting a metabolically favorable microbial configuration in this group.

In the OIS group, five taxa of *Bacteroidota* and *Bacillota* phyla were significantly enriched. These correspond to ASVs belonging to the genera UCG-002, which showed the highest discriminative effect size, followed by the *Eubacterium coprostalinogenes* group, *Bacteroidales*_uncultured, *Veillonella*, and *Intestinibacter*. These taxa are commonly associated with lipid metabolism and bile acid transformation, indicating a distinct functional microbial profile compared to OPS.

The OCG had a single discriminant taxonomic biomarker, *Haemophilus* (*Proteobacteria* phylum), with a high LDA score. The reduced number of enriched taxa in this group suggests a comparatively less differentiated microbial pattern.

## 4. Discussion

Obesity, depression, and anxiety are increasingly recognized as interconnected conditions that share common biological pathways involving chronic low-grade inflammation, gut microbiota dysbiosis, impaired intestinal barrier function, and altered gut–brain axis signaling [1,6,7,8,9,10,11,12,13,14]. Excess adiposity promotes systemic inflammation by releasing pro-inflammatory mediators, while obesity-associated alterations in gut microbiota composition and function may compromise intestinal barrier integrity and facilitate the translocation of lipopolysaccharide (LPS) into circulation [6,9]. This process can amplify immune activation and influence neurobiological pathways involved in mood regulation, including tryptophan metabolism, activation of the kynurenine pathway, and BDNF signaling [11,13,46,47,48]. Consequently, disturbances in microbiota–gut–brain axis homeostasis may contribute simultaneously to metabolic dysfunction and affective symptoms, providing a biological framework for the frequent coexistence of obesity, depression, and anxiety.

In this study, we characterized the taxonomic profile of the gut microbiota in adults with obesity and depressive and/or anxiety symptoms who participated in a structured 12-week weight loss program combining a hypocaloric traditional Mexican diet with moderate-intensity exercise. This WLP led to significant improvements in BC, metabolism, and intestinal permeability, largely independent of baseline emotional status. These clinical benefits were accompanied by selective functional remodeling of the gut microbiota, as no significant changes in overall alpha- and beta-diversity metrics were observed. Notably, the WLP promoted the enrichment of taxa associated with SCFA production, metabolic homeostasis, and gut barrier integrity.

Our findings confirmed that the intervention led to decreases in body weight, BMI, and body fat %, as well as substantial reductions in triglycerides, total cholesterol, VLDL cholesterol, insulin, CRP, liver enzymes (AST, ALT, and GGT), and zonulin. However, all participants remained within the WHO criteria for obesity class I after the intervention. These results align with evidence that lifestyle interventions improve obesity-related metabolic dysfunction through coordinated effects on lipid metabolism, insulin sensitivity, systemic inflammation, and hepatic function [49,50]. The absence of significant group-by-time interactions suggests that metabolic improvements were broadly similar across the three groups. However, baseline differences in bilirubin, albumin, and albumin/globulin ratio indicate heterogeneous hepatic or oxidative stress profiles, particularly in OCG, reflecting distinct underlying metabolic states [51]. Notably, the consistent reduction in CRP across all groups supports a generalized anti-inflammatory effect of the intervention, a key mechanism linking weight reduction to improved metabolic health [52].

We propose that the metabolic regulation observed in our population may be partly explained by the interaction between the TMD, gut microbial metabolism, and exercise. The TMD is naturally rich in polyphenols, fermentable fibers, and microbiota-accessible carbohydrates (MACs), particularly from nixtamalized maize, legumes, prickly pear, chia, tomatoes, squash, and purslane [28]. Along with moderate-intensity exercise, these dietary components may have promoted selective microbial niche remodeling and favored metabolic pathways commonly associated with SCFA production, including acetate, propionate, and butyrate. These metabolites regulate intestinal barrier integrity, immune signaling, appetite control, and systemic inflammation, and also influence neuroplasticity, gene expression, and immune responses; notably, butyrate has been reported to modulate BDNF expression [53,54]. In parallel, previous studies suggest that SCFAs and exercise-induced PGC-1α may influence tryptophan (TRP) metabolism through the kynurenine pathway (KP), representing a potential mechanism linking lifestyle interventions with gut–brain axis regulation [46,47]. Furthermore, SCFAs and bile acids have been reported to influence gut–brain communication through vagal signaling and activation of FXR and TGR5 receptors, respectively [53,55]. However, because none of these metabolites were measured in this cohort, we can only hypothesize rather than demonstrate the role of these mechanisms.

The significant decrease in circulating zonulin after the WLP indicates improved intestinal permeability and has been associated with metabolic regulation. Increased intestinal permeability and elevated zonulin concentrations have been positively correlated with adiposity, insulin resistance, triglycerides, systemic inflammation, neuroinflammation through the KP, endotoxemia, and SCFAs—particularly butyrate and components of metabolic syndrome in humans [56,57]. Therefore, the reduction in zonulin after the WLP suggests that improvements in gut barrier function may have contributed, at least in part, to favorable metabolic outcomes. This effect may be related to exercise- and diet-induced changes in gut microbiota composition and function [58]. However, because microbial metabolites were not measured in this cohort, the specific mechanisms underlying the observed reduction in zonulin remain to be determined.

Although 22% of our population showed improvements in depressive and anxiety symptoms after the WLP, no significant changes were observed in circulating BDNF concentrations. Considering that BDNF signaling is influenced by multiple factors, including age, body weight, stress, physical activity, lifestyle behaviors, and dietary intake [48], this finding is not unexpected and is consistent with the heterogeneous literature on obesity-related lifestyle interventions. For instance, Glud et al. reported significant reductions in circulating BDNF following a 12-week weight loss intervention despite substantial weight loss, with responses varying by sex and intervention modality [58]. In our study, substantial reductions in CRP, insulin, and zonulin indicate improvements in systemic inflammation, insulin sensitivity, and gut barrier function. However, all participants remained within the WHO criteria for class I obesity after the intervention, suggesting the persistence of residual adiposity and obesity-associated inflammation. Therefore, the absence of detectable changes in BDNF may reflect a temporal dissociation between metabolic and neurotrophic adaptations. While metabolic and intestinal improvements can occur within weeks of lifestyle intervention, restoration of neuroplasticity-related pathways may require longer periods of sustained metabolic improvement, inflammatory control, and continued exposure to exercise and dietary interventions.

A major finding of this study was that, according to Wilcoxon, LMM, and LEfSe analyses, the substantial improvements in systemic metabolic markers at the end of the WLP, including CRP, insulin, and zonulin, despite the absence of major changes in alpha and beta diversity and the relative stability in *Bacillota* and *Bacteroidota* abundance, suggest that microbiome-associated benefits may be driven primarily by functional remodeling rather than broad taxonomic restructuring. These results support the increasing evidence that broad diversity indices and the *Bacillota*/*Bacteroidota* ratio are relatively insensitive markers of metabolic health or intervention responsiveness [59]. Similar microbiota stability was previously observed at baseline in this population [32], supporting the existence of a resilient core microbiota, since the diet intervention only modified energy intake rather than altering the habitual dietary components of the traditional Mexican diet. Controlled trials have also shown that dietary interventions often induce functional and taxon-specific microbial shifts without altering overall diversity patterns [60]. Consistent with our findings, Mediterranean diet interventions have been shown to improve metabolic health in individuals with overweight or obesity while promoting enrichment of fiber-degrading and SCFA-associated taxa, despite relatively stable community structure [61]. Similarly, exercise interventions in individuals with depressive symptoms and probiotic supplementation in patients with major depressive disorder have been associated with clinical improvement accompanied by taxon-specific microbial changes rather than global taxonomic reorganization [62,63]. These studies support the concept that microbiota functionality and modulation of specific microbial networks may be more relevant than broad diversity changes in mediating metabolic and affective responses to lifestyle interventions.

Despite this overall compositional stability, significant group-specific microbial changes were identified. The modest increase in *Verrucomicrobiota* observed in OCG is notable, as members of this phylum, especially *Akkermansia muciniphila*, contribute to acetate and propionate production and support butyrate synthesis through cross-feeding interactions, thereby promoting intestinal and metabolic homeostasis [61]. Experimental evidence also suggests that *A. muciniphila*-derived SCFAs may attenuate neuroinflammation and improve depression-like behaviors [64,65].

At the genus level, dominant taxa such as *Prevotella*, *Bacteroides*, *Faecalibacterium*, and *Eubacterium coprostalinogenes* remained relatively stable, as previously reported at baseline [32], supporting the concept of a resilient microbiota structure. However, the OCG showed a dysbiosis-like post-intervention profile, characterized by enrichment of pro-inflammatory *Escherichia*–*Shigella* and *Fusobacterium* as previously reported [32], along with a decrease in butyrate-producing genera, including *Roseburia*, *Ruminococcus*, and *Coprococcus*. The increase in *Escherichia*–*Shigella* should be interpreted cautiously, as transient enrichment may reflect ecological restructuring during dietary adaptation or exercise-induced intestinal physiological changes rather than persistent dysbiosis [66]. Nevertheless, the reduction in butyrate-producing bacteria may have greater biological relevance, given the central role of butyrate in maintaining epithelial integrity and immune homeostasis. Reduced butyrate availability facilitates LPS translocation, producing endotoxemia and consequently chronic low-grade inflammation, key mechanisms linking obesity with systemic dysfunction [59,67]. Although OCG participants did not exhibit depressive or anxiety symptoms, similar microbial configurations under conditions of psychological vulnerability could potentially amplify neuroinflammatory processes associated with mood disorders [68].

In contrast, the OIS group showed enrichment of the *Rikenellaceae* RC9 gut group and *Veillonella*, suggesting enhanced fermentation of MACs and increased lactate utilization, which may promote SCFA production through interconnected metabolic pathways, particularly propionate generation and cross-feeding interactions that support overall microbial function [69]. This finding reflects host–microbial metabolic cooperation associated with improved metabolic flexibility and exercise adaptation. Moderate-intensity exercise may further enhance microbial metabolism by improving intestinal transit and substrate availability [54,70]. Concurrently, the reduction in *Barnesiella* may indicate adaptive reconfiguration of bile acid–microbiota interactions under caloric restriction [71].

Linear mixed model analyses further showed that baseline microbiota composition may partly determine responsiveness to the intervention. The interindividual variability observed in our cohort may also have been influenced by biological sex, as sex hormones, body fat distribution, and immune regulation are recognized modulators of gut microbiota composition and host metabolic and affective phenotypes [72,73]. Although sex was included as a covariate in our analyses, the study was not sufficiently powered to evaluate sex-stratified effects, which should be addressed in future research. Significant group effects for *Actinobacteriota* and *Desulfobacterota* indicate heterogeneous metabolic adaptation among participants [74]. *Actinobacteriota*, particularly *Bifidobacterium*, are promoted by fermentable fibers typical of the TMD and contribute to acetate and lactate production, glucose regulation, and immune homeostasis [75]. In contrast, *Desulfobacterota* are linked to hydrogen sulfide production and pro-inflammatory activity that may compromise gut integrity and reduce beneficial exercise responses [76]. These findings support the idea that baseline microbial ecology may determine responsiveness to dietary and exercise interventions.

At the class level, significant time and interaction effects were observed for *Bacilli* and *Fusobacteriia*. *Bacilli* include taxa such as *Lactobacillus*, which respond positively to dietary fiber and fermented foods common in the TMD [23]. In contrast, *Fusobacteriia* have been associated with inflammatory states and depressive symptomatology, suggesting divergent inflammatory responses among groups [77]. Additional group effects involving *Coriobacteriia*, *Alphaproteobacteria*, *Desulfovibrionia*, and *Actinobacteria* further support the relevance of personalized microbiome responses to dietary interventions [78].

At the genus level, several taxa exhibited significant interaction effects, including *Dorea*, *Streptococcus*, *Bilophila*, *Oscillibacter*, *Megamonas*, *Bifidobacterium*, and *Acidaminococcus*, which are implicated in SCFA production, bile acid transformation, amino acid metabolism, and regulation of the KP [79]. Previous studies have associated *Streptococcus* and *Acidaminococcus* with pro-inflammatory environments and pathways linked to indoleamine 2,3-dioxygenase-1 (IDO1) activation, and kynurenine (KYN) metabolism associated with depressive symptomatology [80]. Conversely, *Bifidobacterium* appears to exert protective effects by preserving systemic TRP availability and reducing the KYN/TRP ratio through anti-inflammatory mechanisms and production of indole derivatives that activate aryl hydrocarbon receptor signaling [81]. Likewise, *Oscillibacter* and *Megamonas* may indirectly regulate KP activity by modulating intestinal permeability and systemic inflammation [82]. Therefore, microbiota-mediated regulation of TRP metabolism occurs primarily through immune–metabolic crosstalk rather than direct substrate conversion. Unfortunately, KYN metabolites were not measured in our cohort.

LEfSe analysis identified twenty-two group-specific microbial signatures among groups after the WLP that also differentiate obese groups according to their affective status as reported before the WLP [32]. This suggests that microbiota responses to combined dietary and exercise interventions depend on the host’s metabolic and affective context. Notably, the OPS group showed limited significant genus-level changes, likely due to reduced statistical power. This pattern may also reflect diminished microbial plasticity or a “resistant microbiome” phenotype associated with persistent metabolic and affective dysfunction [83]. However, this group showed the highest number of discriminant taxa, including enrichment of SCFA-producing genera such as *Barnesiella*, *Bifidobacterium*, *Butyricimonas*, and *Fusicatenibacter*, coexisting with potentially pro-inflammatory microorganisms such as *Porphyromonas*, *Enterococcus*, and *Cronobacter*, as well as lower serum BDNF concentrations. This suggests a functionally heterogeneous ecosystem in which enrichment of SCFA-producing bacteria may not be sufficient to overcome persistent pro-inflammatory signals associated with genera such as *Porphyromonas*, *Enterococcus*, and *Cronobacter*. Therefore, we hypothesize that the persistent pro-inflammatory microbial signature in the OPS group may have limited the functional impact of SCFA-producing taxa, suggesting that restoring microbial functionality alone may be insufficient to improve affective symptoms when residual neuroimmune dysregulation persists. The enrichment of *Porphyromonas* is particularly relevant because this genus has been associated with chronic inflammation, periodontal–gut translocation, neuroinflammation, and immune activation linked to mood disorders and metabolic dysfunction [84]. Experimental evidence indicates that *Porphyromonas gingivalis* may disrupt KYN metabolism through gut–brain axis signaling [85]. Chronic inflammation mediated by LPS and pro-inflammatory cytokines has been shown to suppress BDNF expression and impair neuroplasticity [86]. These findings reinforce the concept that microbiota-mediated affective context outcomes depend on the balance between anti-inflammatory and pro-inflammatory microbial functions, rather than only on the presence of beneficial taxa.

Conversely, the OIS group exhibited a more metabolically favorable microbial profile, characterized by enrichment of UCG-002, *Veillonella*, *Intestinibacter*, and the *Eubacterium coprostalinogenes* group, taxa involved in bile acid metabolism, lipid utilization, and energy harvest. Because bile acids act as signaling molecules through FXR and TGR5 receptors, these microbial adaptations may contribute to improved metabolic and neuroendocrine regulation [86], suggesting that functional optimization of microbial metabolism, rather than increased microbial diversity alone, may underlie improved affective outcomes.

The OCG showed only one discriminant taxon, *Haemophilus*, suggesting that the limited microbial reshaping may reflect partial restoration of eubiosis, given the absence of neuropsychological comorbidities. However, *Haemophilus* has been associated with pro-inflammatory states and mucosal immune activation. Therefore, since our population remained within the WHO criteria for obesity class I after the WLP, its presence may reflect residual low-grade inflammation [87].

The present study also identified specific associations between microbial ASVs and host biochemical and dietary variables, highlighting microbial signatures linked to purine metabolism, liver function, renal physiology, and dietary components. Positive associations between uric acid and *Lactococcus*, *Haemophilus*, *Veillonella*, *Streptococcus*, *Allisonella*, *Romboutsia*, and *Lachnospiraceae* UCG-010 suggest microbial adaptation to metabolic environments characterized by altered purine turnover, oxidative stress, and inflammation [88,89]. In contrast, the negative association between RF39 and uric acid concentrations may reflect a microbial microenvironment associated with beneficial metabolic effects, consistent with previous reports of RF39 enrichment after consumption of fiber-rich foods such as common beans [90].

Associations between *Dialister*, *Lachnospira*, *Lachnospiraceae* UCG-010 with bilirubin markers further support the existence of microbiota–liver metabolic crosstalk. Bilirubin has been recognized for its antioxidant and immunomodulatory functions beyond its role as a hepatic biomarker [91], while gut bacteria participate in enterohepatic metabolic processes and bile acid transformation [92]. Because *Lachnospiraceae* members are major SCFA producers associated with intestinal barrier integrity and anti-inflammatory effects [93,94], their association with bilirubin may reflect coordinated mechanisms of hepatic adaptation and redox regulation.

Another relevant finding was the positive association between *Veillonella* and creatinine concentrations. Although creatinine primarily reflects renal function and muscle metabolism, growing evidence supports bidirectional interactions between kidney physiology and gut microbial ecology [95]. Given *Veillonella’s* ability to metabolize lactate and modulate host energy metabolism, this association may reflect adaptive microbial responses to altered metabolic demand or to exercise-related metabolism [66].

Dietary micronutrient analyses also highlighted the responsiveness of beneficial SCFA-producing taxa to dietary nutrients. The *Eubacterium hallii* group showed positive associations with dietary fiber, potassium, iron, and vitamin A intake, supporting its recognized role in butyrate production and intestinal metabolic homeostasis [96]. Similarly, the association between *Eubacterium siraeum* and dietary cholesterol may be related to microbial responses to dietary lipid exposure and bile acid metabolism [97].

These findings indicate that the beneficial effects of the hypocaloric TMD–exercise intervention are mediated by coordinated host–microbiota interactions involving SCFA production, inflammatory regulation, bile acid metabolism, and intestinal barrier function. Notably, marked improvements in metabolic and inflammatory parameters occurred despite minimal changes in overall microbiota diversity, supporting the concept that microbiota functional adaptations may be more relevant than broad compositional shifts for host metabolic regulation. Consequently, microbiota functionality, rather than diversity alone, may be a more sensitive indicator of responsiveness to lifestyle interventions in obesity. Therefore, this study reinforces the potential of precision nutrition and microbiome-targeted interventions for managing obesity and its emotional comorbidities.

One of the main limitations of this work is that we did not have a non-interventional obese control group. This was due to institutional ethical restrictions designed to protect populations with metabolic vulnerabilities such as obesity, which limits the ability to attribute all observed metabolic and microbial changes exclusively to the WLP. Second, the 12-week intervention period may have been insufficient to induce sustained neurobiological adaptations, as evidenced by detectable changes in circulating BDNF concentrations. Third, all participants remained in WHO grade I obesity after the intervention, suggesting that residual obesity-associated inflammation may have continued to influence gut microbiota composition and intestinal permeability. Fourth, gut microbiota characterization was based on 16S rRNA gene sequencing, which offers limited functional resolution and precludes the direct assessment of microbial metabolic pathways and metabolite production. Consequently, the proposed links between microbiota remodeling and the observed metabolic and affective outcomes, including pathways involving SCFAs, bile acids, kynurenine metabolism, and neurotrophic regulation, should be regarded as biologically plausible hypotheses rather than demonstrated mechanisms since they were not assessed in this cohort. Future studies integrating functional multi-omics approaches are needed to validate these pathways. Fifth, the group sample sizes were unequal because enrollment was voluntary, and participants were included solely on the basis of their willingness to participate in the research, regardless of their baseline mood status.

Despite these limitations, this study provides novel evidence supporting the relevance of microbiota functionality and gut–brain axis interactions in obesity-associated affective phenotypes, and highlights the potential of culturally adapted lifestyle interventions based on the traditional Mexican diet combined with moderate-intensity exercise to promote beneficial metabolic and microbial remodeling.

## 5. Conclusions

The culturally adapted WLP, which combined a hypocaloric traditional Mexican diet with exercise, promoted metabolic, intestinal permeability and BC improvements in all participants and induced selective gut microbiota remodeling characterized by twenty-two group-specific taxonomic biomarkers potentially associated with the improvement and persistence of depressive and anxiety symptoms in the context of obesity.

Although the intervention was not specifically designed to improve mental health outcomes, 22% of participants showed improvements in depressive and anxiety symptoms, and the differential microbial reshaping among groups further indicates that baseline mood status influences microbiota plasticity and responsiveness to the WLP.

Participants with persistent depressive and anxiety symptoms showed the coexistence of SCFA-producing taxa and pro-inflammatory microorganisms, suggesting that chronic low-grade inflammation linked to sustained WHO obesity grade I may reduce the potential neuroprotective effects of beneficial microbial metabolites. In contrast, metabolically favorable microbial signatures enriched in SCFA production and bile acid metabolism were associated with more efficient gut–brain metabolic communication.

Future longitudinal studies integrating metagenomics, metabolomics, and transcriptomics biomarkers are needed to clarify causal relationships and determine whether modulation of microbial pathways can serve as effective therapeutic targets for the obesity–depression–anxiety syndemic.

## Figures and Tables

**Table 1:** Characteristics of the study population.

Groups				
	OCG *n* = 77	OIS *n* = 23	OPS *n* = 6	Total
Males (%)	65 (84.4)	15 (65.2)	3 (50)	83 (78.3)
Females (%)	12 (15.6)	8 (34.8)	3 (50)	23 (21.7)
Age (years) Mean (±SD)	40.1 (7.72)	39.04 (5.69)	33.83 (6.11)	39.44 (7.32)

**Table 2:** Impact of the WLP on body composition, metabolic, neurotrophic, and intestinal permeability biomarkers.

	OCG		OIS		OPS		Group Inter S	Time B vs. A	Interaction GbT
Intervention	Before	After	Before	After	Before	After	*p*-Value	*p*-Value	*p*-Value
Body composition analysis									
Weight (kg)	97.26 (10.94)	85.56 (9.72)	95.58 (11.11)	84.22 (7.88)	98.69 (12.08)	89.33 (9.07)	0.462	**<0.001**	0.920
BMI (kg/m^2^)	34.63 (3.06)	30.47 (2.58)	35.26 (3.34)	31.61 (2.75)	35.81 (4.49)	32 (4.38)	0.287	**<0.001**	0.265
Body Fat (%)	37.40 (5.86)	31.95 (7.51)	39.78 (6.66)	30.83 (7.27)	41.15 (8.20)	29.33 (5.68)	0.863	**<0.001**	0.083
Fat-free mass (%)	62.60 (5.86)	68.14 (7.46)	60.22 (6.66)	69.17 (7.27)	58.85 (8.20)	70.83 (6.014)	0.849	**<0.001**	0.087
Muscle mass (kg)	32.49 (5.24)	31.09 (4.98)	30.48 (6.30)	29.43 (6.11)	31 (2)	29.67 (2.58)	0.306	**<0.001**	0.410
Serum biochemistry									
Glucose (mg/dL)	95.18 (22.79)	89.41 (8.95)	91.45 (13.63)	89.89 (6.80)	85.90 (14.86)	88.10 (8.17)	0.755	0.978	0.834
HDL-cholesterol (mg/dL)	40.42 (9.6)	42.92 (7.75)	43.22 (11.60)	47 (17.33)	38.33 (11.07)	40.17 (10.94)	0.629	0.378	0.528
LDL-cholesterol (mg/dL)	149.85 (177.73)	123.19 (26.96)	137.48 (32.76)	124.22 (27.16)	86.17 (35.25)	96.83 (22.86)	0.691	0.764	0.864
VLDL-cholesterol (mg/dL)	35.61 (18.10)	21.91 (10.28)	32.26 (13.73)	21.87 (8.06)	25.17 (9.8)	19.33 (4.76)	0.812	**0.001**	0.88
Total cholesterol (mg/dL)	206.07 (28.75)	187.99 (33.07)	212.96 (34.95)	190.22 (33.92)	187.17 (80.80)	156.33 (35.57)	0.522	**<0.001**	0.284
TG (mg/dL)	178.41 (90.30)	109.59 (51.60)	163.70 (69.20)	113.17 (35.20)	125.83 (47.90)	96 (24.10)	0.807	**<0.001**	0.835
BUN (mg/dL)	16.09 (20.51)	14.46 (3.10)	22.42 (28.11)	13.25 (4.69)	24 (28.76)	11.63 (3.42)	0.992	**<0.001**	0.944
Creatinine (mg/dL)	0.89 (0.15)	0.92 (0.15)	0.88 (0.16)	0.87 (0.16)	0.83 (0.18)	0.85 (0.025)	0.634	0.197	0.119
CRP (mg/dL)	0.52 (0.67)	0.28 (0.30)	0.76 (1.13)	0.30 (0.22)	0.82 (0.76)	0.52 (0.81)	0.273	**0.006**	0.316
Uric acid (mg/dL)	6.75 (1.62)	6.50 (1.46)	6.52 (1.80)	6.25 (1.67)	6.30 (1.85)	5.38 (1.13)	0.980	**0.034**	0.265
Insulin (Uu/mL)	12.31 (5.92)	5.86 (4.15)	12.70 (4.28)	6.62 (3.59)	13.08 (5.61)	9.03 (9.31)	0.649	**<0.001**	0.750
Cortisol (μg/dL)	8.49 (3.43)	9.31 (2.96)	8.46 (2.44)	8.93 (2.75)	9.91 (2.96)	8.33 (3.40)	0.491	0.984	0.335
Liver panel									
Total Bilirubin (mg/dL)	1.07 (0.38) ^b^	1.08 (0.43)	0.85 (0.26)	0.87 (0.36)	0.78 (0.31)	0.82 (0.34)	**0.006**	0.078	0.833
Direct Bilirubin (mg/dL)	0.26 (1.16)	0.18 (0.15)	0.10 (0.04)	0.18 (0.23)	0.10 (0.10)	0.25 (0.20)	0.737	0.771	0.713
Indirect Bilirubin (mg/dL)	0.97 (0.34) ^b^	0.94 (0.38)	0.75 (0.25)	0.80 (0.29)	0.68 (0.24)	0.72 (0.27)	**0.004**	0.721	0.446
AST (UI/L)	36.66 (28.88)	25.69 (21.67)	27.09 (7.93)	22.55 (4.75)	48.50 (29.79)	24.50 (3.56)	0.146	**<0.001**	0.083
ALT (UI/L)	57.97 (55.92)	33.55 (45.96)	36.05 (22.35)	25.10 (7.34)	81.50 (71.67)	26.95 (9.12)	0.107	**<0.001**	0.085
Total proteins (g/dL)	7.30 (0.034)	6.99 (0.41)	7.26 (0.54)	7.05 (0.39)	7.43 (0.38)	7.05 (0.22)	0.814	**<0.001**	0.500
Albumin (g/dL)	4.33 (0.23) ^b^	4.32 (0.26)	4.18 (0.29)	4.17 (0.28)	4.12 (0.40)	4.05 (0.25)	**0.004**	0.481	0.832
Globulins (g/dL)	2.99 (0.35)	2.66 (0.32)	3.11 (0.32)	2.88 (0.29)	3.32 (0.27)	3 (0.37)	0.054	**<** **0.001**	0.534
Alb/Globulins	1.48 (0.20)	1.78 (1.2)	1.35 (0.15)	1.46 (0.18)	1.25 (0.19)	1.37 (0.25)	**0.001**	0.267	0.686
GGT (UI/L)	45.35 (40.07)	19.74 (11.62)	38.18 (24.58)	20.73 (16.05)	53 (58.56)	16.17 (5.23)	0.633	**<** **0.001**	0.428
ALP (UI/L)	66.11 (16.64)	63.40 (16.79)	68.50 (11.87)	62.07 (14.17)	71 (14.04)	61.32 (9.03)	0.713	**0.003**	0.276
Neurotrophic and intestinal permeability biomarkers									
BDNF (ng/mL)	3.57 (4.33)	3.37 (3.57)	4.93 (6.37)	3.14 (3.54)	2.11 (1.38)	2.49 (1.11)	0.564	0.623	0.555
Zonulin (ng/mL)	53.37 (17.86)	39.51 (16.66)	56.45 (20.04)	36.43 (26.14)	53.64 (26.25)	17.42 (14.95)	0.405	**<0.001**	0.350

OCG; control group of obese participants without symptoms of depression or anxiety before and after the WLP. OIS; group of obese participants who showed improvement in depressive or anxiety symptoms after the WLP. OPS; group of obese participants with persistent depressive or anxiety symptoms after the WLP. Before the WLP intervention, Before; After the WLP intervention, After; Inter Subjects, Inter S; Before vs. After, B vs. A; Group-by-time, GbT; Triglycerides, TG; Blood Urea Nitrogen, BUN; C-Reactive Protein, CRP; Aspartate aminotransferase, AST; Alanine aminotransferase, ALT; Gamma-glutamyl transferase, GGT; Alkaline phosphatase, ALP. Data are mean (±SD). *p*-values are based on the LMM analysis. ^b^: Significant *p*-value obtained from post hoc test of OCG against OPS. Significant values are in bold.

**Table 3:** Effects of the WLP on the Median Relative Abundance at the phylum, class, and genus levels.

Variable	OCG *n* = 70		OIS *n* = 22		OPS *n* = 5		Group Inter S	Time B vs. A	Interaction GbT
	Before	After	Before	After	Before	After	*p*-Value	*p*-Value	*p*-Value
*Bacillota*/*Bacteroidota*	0.77 (0.70)	1.3 (3.26)	0.81 (0.76)	1.54 (2.79)	1.03 (0.74)	0.87 (0.52)	0.681	0.353	0.749
*Bacteroidota*	48.85 (23.27)	46.68 (22.08)	46.64 (25.16)	40.45 (24.16)	36.29 (20.56)	37.66 (20.60)	0.477	0.458	0.727
*Bacillota*	34.62 (19.78)	38.57 (21.13)	33.76 (20.39)	40.60 (24.93)	40.18 (23.36)	37.66 (20.29)	0.720	0.584	0.593
*Proteobacteria*	3.40 (6.84)	3.60 (5.08)	5.21 (9.04)	4.23 (6.49)	3.46 (4.92)	5.60 (9.33)	0.520	0.492	0.649
*Verrucomicrobiota*	0.25 (0.83)	1.02 (3.04)	0.47 (1.03)	0.42 (1.21)	0.14 (0.16)	0.64 (0.92)	0.534	0.346	0.362
*Actinobacteriota*	0.51 (0.66)	0.38 (0.48)	0.41 (0.62)	0.49 (0.73)	2.18 (3.92)	1.40 (1.14)	**0.005**	0.094	0.223
*Desulfobacterota*	0.41 (0.51)	0.40 (0.71)	0.31 (0.44)	0.42 (0.71)	0.99 (0. 84)	0.41 (0.46)	**0.030**	0.257	0.148
*Cyanobacteria*	0.27 (0.93)	0.28 (0.68)	0.09 (0.27)	0.16 (0.33)	0.46 (0.87)	0.30 (0.64)	0.480	0.848	0.877
*Spirochaetota*	0.00 (0.01)	0.04 (0.42)	0.00 (0.01)	0.27 (0.87)	0.00 (0.00)	0.00 (0.00)	0.813	0.246	0.210
*Fusobacteriota*	0.02 (0.05)	0.02 (0.05)	0.09 (0.23)	0.00 (0.01)	0.00 (0.00)	0.00 (0.01)	0.066	0.131	**0.018**
*Bacteroidia*	48.85 (23.27)	46.68 (22.08)	46.64 (25.16)	40.45 (24.16)	36.29 (20.56)	37.66 (20.60)	0.477	0.458	0.727
*Clostridia*	30.40 (18.57)	34.08 (20.79)	28.99 (18.61)	36.17 (23.82)	36.80 (21.49)	32.33 (16.58)	0.645	0.710	0.463
*Bacilli*	1.08 (1.14)	1.01 (1.09)	0.89 (1.17)	2.18 (3.99)	1.73 (1.47)	2.84 (2.88)	0.220	**0.048**	**0.008**
*Gammaproteobacteria*	3.33 (6.85)	3.44 (5.01)	5.18 (9.04)	4.18 (6.48)	3.13 (4.84)	5.55 (9.21)	0.525	0.469	0.625
*Negativicutes*	3.11 (3.45)	3.42 (4.73)	3.86 (3.77)	2.21 (2.58)	1.55 (1.60)	2.27 (2.94)	0.408	0.857	0.229
*Verrucomicrobiae*	0.21 (0.81)	0.90 (2.90)	0.42 (1.01)	0.34 (1.14)	0.08 (0.11)	0.47 (0.84)	0.512	0.333	0.353
*Desulfovibrionia*	0.36 (0.50)	0.36 (0.68)	0.27 (0.42)	0.37 (0.67)	0.82 (0.85)	0.34 (0.45)	**0.030**	0.258	0.148
*Coriobacteriia*	0.29 (0.40)	0.28 (0.44)	0.23 (0.33)	0.37 (0.66)	0.98 (1.90)	0.78 (0.75)	**0.013**	0.661	0.316
*Vampirivibrionial*	0.27 (0.93)	0.28 (0.68)	0.09 (0.27)	0.16 (0.33)	0.46 (0.87)	0.30 (0.64)	0.480	0.848	0.877
*Alphaproteobacteria*	0.07 (0.26)	0.15 (0.58)	0.036 (0.15)	0.04 (0.15)	0.32 (0.79)	0.05 (0.12)	**0.046**	0.495	0.070
*Actinobacteria*	0.16 (0.32)	0.060 (0.11)	0.12 (0.35)	0.050 (0.10)	0.82 (1.72)	0.38 (0.43)	**0.005**	**0.007**	0.169
*Spirochaetia*	0.00 (0.01)	0.05 (0.42)	0.00 (0.02)	0.26 (0.87)	0.00 (0.00)	0.00 (0.00)	0.838	0.258	0.238
*Fusobacteriia*	0.02 (0.05)	0.02 (0.05)	0.09 (0.23)	0.01 (0.02)	0.01 (0.01)	0.01 (0.01)	0.066	0.131	**0.018**
*Prevotella*	24.16 (22.33)	20.89 (20.12)	22.67 (23.48)	19.45 (19.14)	9.90 (8.28)	14.41 (10.58)	0.708	0.923	0.874
*Bacteroides*	13.47 (16.35)	12.97 (17.24)	13.41 (14.72)	10.37 (16.86)	13.26 (15.08)	7.72 (6.52)	0.680	0.129	0.830
*Faecalibacterium*	5.37 (5.22)	6.5 (10.36)	5.33 (4.42)	8.15 (11.45)	5.65 (4.21)	6.26 (3.57)	0.964	0.670	0.556
*UCG002*	3.92 (4.12)	6.29 (10.40)	3.56 (3.14)	4.04 (2.64)	5.34 (4.90)	3.75 (2.40)	0.603	0.538	0.263
*Eubacterium coprostanoligenes*	2.66 (2.92)	2.90 (4.11)	2.16 (2.28)	1.50 (2.05)	2.92 (2.95)	4.97 (8.67)	0.711	0.225	0.394
*Escherichia-Shigella*	1.58 (2.32)	1.91 (2.26)	5.19 (8.88)	3.90 (4.47)	0.82 (1.10)	2.60 (2.92)	**0.004**	0.762	0.351
*Alistipe*	2.43 (3.51)	1.96 (1.22)	1.61 (2.41)	2.15 (1.47)	2.84 (3.44)	2.61 (1.56)	0.311	0.618	0.204
*Roseburia*	2.14 (2.82)	1.80 (3.61)	2.06 (2.90)	2.82 (4.63)	1.99 (1.57)	1.12 (1.77)	0.916	0.562	0.397
*Ruminococcus*	1.99 (1.95)	1.90 (4.81)	1.74 (1.85)	1.83 (3.76)	2.14 (2.90)	1.48 (0.97)	0.792	0.626	0.927
*Lachnospiraceae NK4A136*	1.56 (2.05)	1.78 (1.19)	2.49 (4.14)	1.88 (2.04)	1.07 (0.99)	2.44 (3.15)	0.267	0.614	0.239
*Parabacteroides*	1.59 (1.57)	1.97 (2.40)	1.27 (1.08)	1.37 (1.44)	1.60 (0.92)	1.42 (1.23)	0.514	0.878	0.845
*Succinivibrio*	1.76 (6.10)	1.96 (4.48)	0.52 (0.88)	1.08 (1.88)	0.56 (0.64)	0.65 (1.32)	0.607	0.807	0.947
*Muribaculaceae*	1.42 (2.83)	1.16 (1.41)	1.71 (2.83)	1.43 (1.88)	0.21 (0.51)	0.71 (0.60)	0.418	0.913	0.767
*Alloprevotella*	1.33 (1.90)	1.30 (2.96)	1.66 (2.43)	0.87 (0.98)	0.22 (0.24)	0.95 (1.81)	0.214	0.732	0.435
*Eubacterium siraeum group*	1.22 (1.87)	1.29 (3.09)	1.10 (1.26)	0.71 (1.63)	1.21 (1.24)	1.14 (2.20)	0.907	0.833	0.598
*Coprococcus*	1.28 (1.50)	0.82 (1.82)	0.69 (0.89)	2.36 (5.19)	1.22 (0.97)	0.41 (0.41)	0.218	0.676	**0.011**
*Barnesiella*	0.71 (1.82)	0.99 (3.68)	1.15 (3.91)	0.88 (3.88)	4.00 (4.96)	3.65 (8.83)	**0.020**	0.628	0.978
*Subdoligranulum*	0.85 (1.63)	1.16 (1.49)	1.00 (1.62)	1.30 (1.60)	1.50 (1.37)	1.46 (1.00)	0.064	0.652	0.523
*Dialister*	1.03 (2.79)	1.16 (1.84)	1.23 (2.08)	1.10 (3.58)	0.26 (0.40)	0.99 (1.29)	0.873	0.569	0.900
*Ruminococcus torques group*	0.81 (1.46)	0.92 (1.37)	0.98 (1.31)	1.00 (1.59)	1.07 (0.74)	1.47 (2.87)	0.984	0.878	0.579
*Dorea*	0.59 (1.40)	0.80 (0.72)	0.58 (0.64)	1.06 (1.02)	4.49 (5.73)	0.79 (0.48)	**<0.001**	**0.004**	**<0.001**
*Clostridia UCG014*	0.62 (0.67)	0.77 (0.83)	0.98 (1.38)	0.87 (1.02)	0.61 (0.44)	0.69 (0.75)	0.207	0.794	0.601
*UCG003*	0.64 (0.66)	0.72 (1.12)	0.64 (0.46)	0.33 (0.55)	1.10 (1.11)	3.07 (5.04)	0.398	**0.016**	**0.044**
*RikenellaceaeRC9*	0.74 (1.83)	0.55 (0.70)	0.21 (0.30)	1.16 (1.47)	0.65 (0.91)	2.15 (2.76)	0.356	**0.030**	**0.011**
*UCG005*	0.61 (0.62)	0.70 (0.63)	0.44 (0.31)	0.77 (0.55)	0.73 (0.62)	0.61 (0.23)	0.414	0.473	0.301
*NK4A21 group*	0.52 (0.57)	0.76 (1.56)	0.56 (0.48)	0.44 (0.92)	1.03 (1.22)	0.70 (0.73)	0.284	0.660	0.748
*Paraprevotella*	0.52 (0.70)	0.72 (2.23)	0.56 (1.09)	0.24 (0.50)	0.89 (0.53)	1.24 (2.02)	0.810	0.833	0.623
*Phascolarctobacterium*	0.49 (0.57)	0.61 (0.53)	0.78 (1.27)	0.75 (1.04)	0.41 (0.29)	0.52 (0.42)	0.330	0.921	0.882
*uncultured X*	0.55 (0.46)	0.55 (0.88)	0.48 (0.42)	0.65 (0.70)	0.42 (0.46)	0.59 (0.36)	0.530	0.609	0.582
*Blautia*	0.49 (0.98)	0.55 (0.73)	0.41 (0.53)	0.66 (0.73)	0.64 (0.84)	0.39 (0.20)	0.892	0.814	0.649
*Veillonella*	0.52 (1.19)	0.48 (0.40)	0.21 (0.40)	0.54 (0.61)	0.25 (0.36)	0.76 (0.14)	0.600	0.097	0.427
*Christensenellaceae R7 group*	0.39 (0.55)	0.48 (0.46)	0.38 (0.53)	0.46 (0.35)	1.14 (1.56)	0.60 (0.40)	0.136	0.134	0.331
*Akkermansia*	0.37 (0.88)	0.39 (1.27)	0.51 (1.07)	0.85 (1.75)	0.23 (0.28)	0.14 (0.18)	0.768	0.749	0.765
*UCG010*	0.39 (0.42)	0.44 (0.60)	0.38 (0.35)	0.51 (0.84)	0.56 (0.28)	0.59 (0.49)	0.827	0.623	0.909
*Gastranaerophilales*	0.41 (1.58)	0.43 (0.58)	0.087 (0.29)	0.36 (0.59)	0.61 (0.93)	0.34 (0.47)	0.490	0.961	0.641
*Haemophilus*	0.41 (0.84)	0.36 (0.49)	0.10 (0.13)	0.43 (0.74)	0.31 (0.33)	0.75 (0.46)	0.232	0.084	0.286
*Streptococcus*	0.35 (0.45)	0.28 (0.57)	0.27 (0.60)	0.39 (0.57)	0.23 (0.11)	1.56 (1.72)	0.722	**0.002**	**0.001**
*Catenibacterium*	0.30 (0.38)	0.29 (0.34)	0.30 (0.43)	0.25 (0.33)	0.58 (0.83)	0.98 (1.60)	0.414	0.148	0.318
*Desulfovibrio*	0.34 (0.50)	0.35 (1.43)	0.21 (0.28)	0.26 (0.54)	0.08 (0.09)	0.007 (0.01)	0.202	0.831	0.957
*Butyrivibrio*	0.30 (0.71)	0.30 (0.64)	0.37 (0.87)	0.37 (0.64)	0.02 (0.04)	0.29 (0.45)	0.685	0.489	0.895
*Sutterella*	0.29 (0.36)	0.29 (0.24)	0.29 (0.33)	0.30 (0.37)	0.34 (0.43)	0.38 (0.18)	0.982	0.769	0.911
*Monoglobus*	0.21 (0.38)	0.29 (0.55)	0.52 (1.09)	0.31 (0.45)	0.42 (0.61)	0.20 (0.17)	0.216	0.260	0.435
*Clostridium sensu stricto1*	0.28 (0.74)	0.31 (0.71)	0.079 (0.13)	0.19 (0.35)	1.14 (0.73)	0.36 (0.70)	**0.003**	0.216	0.146
*Collinsella*	0.20 (0.55)	0.28 (0.74)	0.37 (0.97)	0.17 (0.34)	0.76 (1.56)	0.44 (0.91)	0.149	0.481	0.392
*Odoribacter*	0.24 (0.22)	0.26 (0.48)	0.22 (0.38)	0.30 (0.86)	0.36 (0.38)	0.097 (0.14)	0.746	0.335	0.641
*Butyricimonas*	0.20 (0.30)	0.26 (0.30)	0.36 (0.76)	0.15 (0.16)	0.35 (0.26)	0.43 (0.24)	0.291	0.881	0.120
*Bifidobacterium*	0.20 (0.46)	0.24 (0.44)	0.088 (0.11)	0.19 (0.25)	0.92 (1.91)	0.23 (0.25)	**0.008**	0.318	**0.020**
*Lachnospiraceae UCG004*	0.21 (0.29)	0.21 (0.33)	0.24 (0.26)	0.21 (0.29)	0.20 (0.32)	0.40 (0.33)	0.930	0.378	0.632
*Eubacterium ruminantium group*	0.20 (0.36)	0.16 (0.23)	0.23 (0.41)	0.40 (0.88)	0.11 (0.07)	0.15 (0.14)	0.747	0.755	0.256
*Megasphaera*	0.21 (0.54)	0.13 (0.33)	0.21 (0.63)	0.52 (1.15)	0.09 (0.18)	0.10 (0.14)	0.909	0.558	0.057
*Eubacterium eligens group*	0.21 (0.38)	0.21 (0.57)	0.13 (0.14)	0.11 (0.20)	0.11 (0.08)	0.45 (0.70)	0.748	0.358	0.463
*Holdemanella*	0.15 (0.22)	0.23 (0.67)	0.15 (0.22)	0.11 (0.36)	0.44 (0.75)	0.15 (0.21)	0.105	0.268	0.685
*Eubacterium xylanophilum group*	0.16 (0.64)	0.18 (0.31)	0.21 (0.52)	0.27 (0.35)	0.06 (0.08)	0.19 (0.40)	0.825	0.726	0.810
*Lachnoclostridium*	0.14 (0.33)	0.19 (0.26)	0.20 (0.34)	0.19 (0.32)	0.18 (0.15)	0.16 (0.15)	0.784	0.975	0.941
*Lachnospiraceae UCG001*	0.16 (0.36)	0.20 (0.25)	0.05 (0.08)	0.14 (0.19)	0.46 (0.84)	0.14 (0.18)	**0.050**	0.780	**0.042**
*RF39*	0.15 (0.19)	0.16 (0.35)	0.12 (0.19)	0.17 (0.30)	0.13 (0.11)	0.39 (0.36)	0.824	0.182	0.267
*Acidaminococcus*	0.14 (0.28)	0.13 (0.20)	0.05 (0.09)	0.27 (0.64)	0.15 (0.17)	0.50 (0.64)	0.295	**0.035**	**0.023**
*Romboutsia*	0.10 (0.13)	0.001 (0.009)	0.16 (0.33)	0.80 (3.58)	0.14 (0.09)	0.00 (0.01)	0.432	0.755	0.135
*Comamonas*	0.08 (0.16)	0.15 (0.28)	0.22 (0.83)	0.19 (0.38)	0.04 (0.06)	0.05 (0.04)	0.433	0.923	0.839
*Allisionella*	0.13 (0.22)	0.16 (0.20)	0.06 (0.05)	0.12 (0.15)	0.08 (0.09)	0.17 (0.10)	0.453	0.205	0.898
*Citrobacter*	0.14 (0.36)	0.15 (0.26)	0.03 (0.06)	0.13 (0.23)	0.02 (0.05)	0.14 (0.12)	0.323	0.300	0.610
*Anaerostipes*	0.11 (0.17)	0.16 (0.60)	0.07 (0.11)	0.05 (0.16)	0.22 (0.19)	0.06 (0.13)	0.185	0.789	0.571
*Parasutterella*	0.13 (0.51)	0.10 (0.17)	0.07 (0.18)	0.22 (0.59)	0.01 (0.02)	0.03 (0.04)	0.707	0.705	0.370
*Lachnospira*	0.10 (0.16)	0.11 (0.12)	0.10 (0.17)	0.17 (0.20)	0.09 (0.05)	0.09 (0.05)	0.911	0.595	0.413
*Paraprevotellaceae NK3B31 group*	0.12 (0.23)	0.05 (0.44)	0.03 (0.07)	0.30 (0.93)	0.05 (0.00)	0.00 (0.00)	0.140	0.535	0.117
*Erysipelotrichaceae UCG003*	0.08 (0.22)	0.11 (0.54)	0.04 (0.07)	0.02 (0.04)	0.34 (0.66)	0.01 (0.02)	0.068	0.394	0.213
*Bilophila*	0.05 (0.21)	0.11 (0.38)	0.10 (0.21)	0.03 (0.08)	0.57 (1.16)	0.02 (0.04)	**0.005**	**0.016**	**0.037**
*Eubacterium ventriossum group*	0.08 (0.11)	0.01 (0.050)	0.087 (0.16)	0.38 (1.56)	0.14 (0.20)	0.02 (0.06)	0.960	0.971	0.088
*Klebsiella*	0.08 (0.55)	0.07 (0.15)	0.058 (0.16)	0.08 (0.10)	0.00 (0.00)	0.16 (0.16)	0.969	0.432	0.890
*Slackia*	0.07 (0.21)	0.08 (0.23)	0.02 (0.03)	0.09 (0.16)	0.28 (0.39)	0.05 (0.01)	0.053	0.324	0.149
*Oscillibacter*	0.08 (0.10)	0.05 (0.12)	0.05 (0.07)	0.05 (0.10)	0.03 (0.02)	0.44 (0.42)	0.508	**<0.001**	**<0.001**
*Mitsuokella*	0.05 (0.09)	0.08 (0.16)	0.08 (0.14)	0.04 (0.08)	0.07 (0.11)	0.10 (0.10)	0.422	0.653	0.213
*Weissella*	0.05 (0.16)	0.09 (0.41)	0.06 (0.17)	0.00 (0.01)	0.02 (0.03)	0.04 (0.10)	0.738	0.857	0.698
*Clostridiavadin BB60*	0.05 (0.11)	0.03 (0.13)	0.06 (0.09)	0.14 (0.40)	0.02 (0.01)	0.13 (0.21)	0.477	0.194	0.140
*Prevotellaceae UCG003*	0.03 (0.13)	0.07 (0.22)	0.11 (0.37)	0.03 (0.07)	0.00 (0.00)	0.03 (0.05)	0.225	0.938	0.275
*Ruminococcus navus group*	0.03 (0.10)	0.047 (0.10)	0.08 (0.13)	0.09 (0.29)	0.00 (0.01)	0.025 (0.04)	0.254	0.974	0.854
*Family XII UCG 001*	0.04 (0.08)	0.041 (0.15)	0.03 (0.07)	0.01 (0.03)	0.03 (0.05)	0.26 (0.57)	0.958	**0.020**	0.083
*Lachnospiraceae UCG003*	0.04 (0.14)	0.02 (0.08)	0.00 (0.02)	0.12 (0.53)	0.00 (0.00)	0.00 (0.00)	0.508	0.657	0.117
*Fusobacterium*	0.02 (0.10)	0.03 (0.06)	0.05 (0.22)	0.04 (0.05)	0.00 (0.01)	0.04 (0.08)	0.565	0.917	.0586
*Lactobacillus*	0.01 (0.06)	0.01 (0.08)	0.00 (0.00)	0.05 (0.23)	0.1 (0.12)	0.00 (0.00)	**0.005**	0.469	0.100
*Anaerovibrio*	0.01 (0.05)	0.01 (0.04)	0.00 (0.01)	0.06 (0.13)	0.00 (0.00)	0.00 (0.00)	0.697	0.117	**0.026**
*Hungatella*	0.01 (0.04)	0.03 (0.18)	0.01 (0.05)	0.00 (0.01)	0.00 (0.0)	0.00 (0.00)	0.771	0.977	0.817
*Raoultella*	0.00 (0.03)	0.028 (0.15)	0.00 (0.00)	0.00 (0.00)	0.07 (0.15)	0.00 (0.00)	**0.012**	0.501	0.461
*Tyzzerella*	0.01 (0.02)	0.02 (0.05)	0.00 (0.01)	0.01 (0.02)	0.00 (0.00)	0.01 (0.01)	0.782	0.331	0.770
*Megamonas*	0.00 (0.01)	0.01 (0.03)	0.00 (0.01)	0.01 (0.03)	0.00 (0.0)	0.09 (0.21)	0.937	**0.001**	**0.005**
*Treponema*	0.00 (0.00)	0.00 (0.00)	0.00 (0.02)	0.05 (0.23)	0.01 (0.02)	0.00 (0.00)	**0.048**	0.303	0.266
*Asteroleplasma*	0.00 (0.00)	0.01 (0.02)	0.00 (0.00)	0.02 (0.04)	0.00 (0.00)	0.00 (0.01)	0.782	**0.022**	0.198
*Paeniclostridium*	0.00 (0.00)	0.00 (0.03)	0.00 (0.00)	0.00 (0.00)	0.00 (0.00)	0.00 (0.01)	0.998	0.706	0.896

The sample size (n) for each group was adjusted to account for missing microbiota data. Data are presented as mean (±SD). *p*-values are based on LMM analysis. Bold values indicate results that remained statistically significant after FDR correction for multiple testing. OCG; control group of obese participants without symptoms of depression or anxiety before and after the WLP. OIS; group of obese participants who showed improvement in depressive or anxiety symptoms after the WLP. OPS; group of obese participants with persistent depressive or anxiety symptoms after the WLP. Before the WLP intervention, Before; After the WLP intervention, After; Inter Subjects, Inter S; Before vs. After, B vs. A; Group-by-time, GbT.

## Data Availability

The datasets generated and/or analyzed are available from the corresponding author. The raw data have been submitted to the NCBI platform and are publicly available at BioProject ID: PRJNA1289340. The original data presented in the study are openly available in the NCBI’s Sequence Read Archive (SRA) at [http://www.ncbi.nlm.nih.gov/bioproject/1289340] or [accession number PRJNA1289340] (accessed on 9 July 2025).

## Abbreviations

ALT	Alanine aminotransferase
AST	Aspartate aminotransferase
ASVs	Amplicon sequence variants
BAI	Beck Anxiety Inventory
BDI	Beck Depression Inventory
BC	Body composition
BMI	Body mass index
BNDF	Brain-derived neurotrophic factor
CRP	C-reactive protein
FDR	False discovery rate
GGT	Gamma-Glutamyl Transferase
HPA	Hypothalamic–pituitary–adrenal
KYNA	Kynurenic acid
KYN	Kynurenine
KP	Kynurenine pathway
LMMs	Linear Mixed-Effects Models
LEfSe	Linear discriminant effect size
MACs	Microbiota-accessible carbohydrates
MRA	Median relative abundance
OCG	Group Control without symptoms
OIS	Group with improved symptoms
OPS	Group with persistent symptoms
OTUs	Operational taxonomic units
SCFAs	Short-chain fatty acids
SD	Standard deviation
TMD	Traditional Mexican diet
TRP	Tryptophan
WLP	Weight loss program

## Appendix A

**Table A1:** Baseline and Final Nutrient Intake.

Time Point	Energy Kcal	Prot (g)	Lip (g)	CH (g)	SFA (g)	MFA (g)	PFA (g)	Chol (mg)	Sugar (g)	Fiber (g)	Vit A (µg RE)	Ca (mg)	Iron (mg)	Potassium (mg)	Sodium (mg)
Basal	2133	112	70	246	19	20	9	614	37	14	554	842	26	1496	1834
Final	1426	90	40	176	10	12	6	329	28	24	1057	914	38	2591	1133

Average daily nutrient intake of all participants. Protein, Prot; Lipids, Lip; Carbohydrates, CH; Saturated fatty acids, SFA; Monosaturated fatty acids, MFA; Polyunsaturated fatty acids, PFA; Cholesterol, Chol; Vitamin A, Vit A; Calcium, Ca.

## Appendix C

**Table A2:** Median relative abundance of phylum, class, and genera before and after the WLP.

	OCG (*n* = 70)		OIS (*n* = 20)		OPS (*n* = 5)	
Variable	Before	After	Before	After	Before	After
*Bacillota*/*Bacteroidota*	0.88 (0.69)	1.53 (3.39)	0.93 (0.75)	1.78 (2.93)	1.24 (0.62)	1.05 (0.34)
*Bacteroidota*	55.32 (15.86)	51.36 (17.15)	53.65 (18.39)	46.52 (19.47)	43.55 (11.57)	45.2 (10.25)
*Bacillota*	39.21 (16.17)	42.43 (18.05)	38.83 (16.57)	46.7 (20.51)	48.22 (14.07)	45.2 (9.45)
*Proteobacteria*	3.86 (7.17)	3.97 (5.2)	6 (9.47)	4.86 (6.75)	4.16 (5.17)	6.72 (9.98)
*Verrucomicrobiota*	0.29 (0.88)	1.02 (3.04)	0.54 (1.09)	0.42 (1.22)	0.17 (0.17)	0.64 (0.92)
*Actinobacteriota*	0.52 (0.67)	0.38 (0.48)	0.42 (0.62)	0.49 (0.74)	2.18 (3.93)	1.4 (1.14)
*Desulfobacterota*	0.42 (0.51)	0.4 (0.71)	0.31 (0.44)	0.43 (0.71)	0.99 (0.85)	0.41 (0.47)
*Cyanobacteria*	0.32 (0.99)	0.31 (0.72)	0.1 (0.29)	0.19 (0.35)	0.56 (0.95)	0.37 (0.7)
*Spirochaetota*	0 (0.02)	0.05 (0.44)	0.01 (0.02)	0.31 (0.93)	0 (0)	0 (0)
*Fusobacteriota*	0.02 (0.06)	0.03 (0.06)	0.1 (0.25)	0.01 (0.02)	0.01 (0.01)	0.01 (0.01)
*Bacteroidia*	55.32 (15.86)	51.36 (17.15)	53.65 (18.39)	46.52 (19.47)	43.55 (11.57)	45.2 (10.25)
*Clostridia*	34.43 (15.84)	37.49 (18.61)	33.34 (15.76)	41.6 (20.49)	44.17 (13.09)	38.8 (5.51)
*Gammaproteobacteria*	3.77 (7.18)	3.79 (5.14)	5.96 (9.48)	4.82 (6.75)	3.77 (5.14)	6.66 (9.84)
*Negativicutes*	3.53 (3.47)	3.77 (4.84)	4.44 (3.72)	2.55 (2.61)	1.87 (1.58)	2.72 (3.05)
*Bacilli*	1.23 (1.14)	1.11 (1.1)	1.03 (1.21)	2.51 (4.19)	2.08 (1.35)	3.41 (2.83)
*Desulfovibrionia*	0.42 (0.51)	0.4 (0.71)	0.31 (0.44)	0.43 (0.71)	0.99 (0.85)	0.41 (0.47)
*Coriobacteriia*	0.33 (0.42)	0.31 (0.46)	0.28 (0.35)	0.44 (0.7)	1.19 (2.06)	0.94 (0.73)
*Vampirivibrionia*	0.32 (0.99)	0.31 (0.72)	0.1 (0.29)	0.19 (0.35)	0.56 (0.95)	0.37 (0.7)
*Verrucomicrobiae*	0.24 (0.86)	0.99 (3.04)	0.49 (1.08)	0.39 (1.23)	0.1 (0.11)	0.56 (0.91)
*Actinobacteria*	0.18 (0.34)	0.07 (0.12)	0.14 (0.38)	0.06 (0.11)	0.99 (1.87)	0.46 (0.44)
*Alphaproteobacteria*	0.08 (0.28)	0.17 (0.62)	0.04 (0.16)	0.05 (0.16)	0.39 (0.87)	0.06 (0.14)
*Fusobacteriia*	0.02 (0.06)	0.03 (0.06)	0.1 (0.25)	0.01 (0.02)	0.01 (0.01)	0.01 (0.01)
*Spirochaetia*	0 (0.02)	0.05 (0.44)	0.01 (0.02)	0.3 (0.93)	0 (0)	0 (0)
*Prevotella*	27.36 (21.84)	22.99 (19.94)	26.08 (23.35)	22.37 (18.86)	11.88 (7.52)	17.3 (8.82)
*Bacteroides*	15.26 (16.6)	14.27 (17.57)	15.43 (14.78)	11.93 (17.6)	15.92 (15.22)	9.27 (5.94)
*Faecalibacterium*	6.09 (5.15)	7.2 (10.66)	6.13 (4.19)	9.38 (11.82)	6.79 (3.55)	7.51 (2.06)
*UCG002*	4.44 (4.12)	6.93 (10.72)	4.1 (3.02)	4.65 (2.27)	6.41 (4.64)	4.51 (1.74)
*Eubacterium coprostanoligenes group*	3.02 (2.93)	3.19 (4.21)	2.49 (2.28)	1.74 (2.11)	3.52 (2.88)	5.97 (9.3)
*Alistipes*	2.76 (3.62)	2.16 (1.1)	1.86 (2.5)	2.48 (1.3)	3.41 (3.53)	3.14 (1.01)
*Roseburia*	2.43 (2.89)	1.99 (3.74)	2.38 (3)	3.25 (4.84)	2.4 (1.37)	1.35 (1.89)
*Ruminococcus*	2.26 (1.94)	2.1 (5.01)	2 (1.85)	2.11 (3.98)	2.58 (3.03)	1.78 (0.73)
*Succinivibrio*	2 (6.47)	2.16 (4.67)	0.61 (0.93)	1.24 (1.98)	0.67 (0.65)	0.79 (1.44)
*Parabacteroides*	1.81 (1.56)	2.18 (2.44)	1.46 (1.03)	1.58 (1.44)	1.92 (0.56)	1.71 (1.14)
*Escherichia-Shigella*	1.79 (2.4)	2.11 (2.29)	5.98 (9.3)	4.49 (4.52)	0.99 (1.14)	3.12 (2.94)
*Lachnospiraceae NK4A136 group*	1.74 (2.1)	1.97 (1.11)	2.87 (4.33)	2.16 (2.05)	1.29 (0.95)	2.93 (3.26)
*Muribaculaceae*	1.61 (2.96)	1.28 (1.43)	1.97 (2.96)	1.65 (1.93)	0.26 (0.56)	0.86 (0.56)
*Alloprevotella*	1.51 (1.97)	1.44 (3.08)	1.92 (2.52)	1.01 (1)	0.27 (0.24)	1.15 (1.96)
*Coprococcus*	1.46 (1.52)	0.9 (1.89)	0.8 (0.91)	2.73 (5.5)	1.48 (0.86)	0.5 (0.4)
*Eubacterium siraeum group*	1.39 (1.93)	1.42 (3.22)	1.27 (1.28)	0.82 (1.73)	1.46 (1.22)	1.37 (2.39)
*Dialister*	1.03 (2.8)	1.16 (1.85)	1.23 (2.09)	1.11 (3.58)	0.26 (0.4)	1 (1.29)
*Subdoligranulum*	0.97 (1.71)	1.16 (1.49)	1.15 (1.7)	1.3 (1.61)	1.81 (1.29)	1.46 (1)
*Ruminococcus torques group*	0.82 (1.47)	0.93 (1.37)	0.98 (1.32)	1.01 (1.59)	1.08 (0.75)	1.47 (2.88)
*Barnesiella*	0.81 (1.93)	1.09 (3.85)	1.33 (4.19)	1.01 (4.16)	4.8 (5.1)	4.38 (9.67)
*Rikenellaceae RC9 gut group*	0.74 (1.83)	0.55 (0.71)	0.21 (0.31)	1.16 (1.48)	0.65 (0.91)	2.15 (2.77)
*UCG003*	0.64 (0.67)	0.73 (1.13)	0.64 (0.46)	0.33 (0.55)	1.11 (1.11)	3.07 (5.04)
*Clostridia UCG014*	0.62 (0.68)	0.78 (0.84)	0.99 (1.39)	0.87 (1.03)	0.62 (0.44)	0.69 (0.76)
*UCG005*	0.62 (0.62)	0.7 (0.63)	0.44 (0.31)	0.77 (0.56)	0.73 (0.63)	0.61 (0.23)
*Dorea*	0.6 (1.4)	0.81 (0.73)	0.58 (0.65)	1.07 (1.03)	4.49 (5.73)	0.79 (0.48)
*Paraprevotella*	0.53 (0.71)	0.72 (2.24)	0.56 (1.09)	0.24 (0.5)	0.89 (0.53)	1.25 (2.03)
*Veillonella*	0.53 (1.2)	0.49 (0.41)	0.21 (0.4)	0.55 (0.62)	0.26 (0.37)	0.77 (0.14)
*NK4A214 group*	0.52 (0.57)	0.76 (1.56)	0.57 (0.48)	0.44 (0.92)	1.03 (1.22)	0.71 (0.74)
*Blautia*	0.49 (0.99)	0.55 (0.73)	0.41 (0.54)	0.67 (0.73)	0.64 (0.85)	0.39 (0.21)
*Phascolarctobacterium*	0.49 (0.57)	0.61 (0.54)	0.79 (1.28)	0.76 (1.04)	0.41 (0.3)	0.53 (0.42)
*Gastranaerophilales*	0.42 (1.58)	0.43 (0.58)	0.09 (0.29)	0.36 (0.59)	0.62 (0.94)	0.35 (0.48)
*Haemophilus*	0.42 (0.84)	0.36 (0.49)	0.1 (0.14)	0.44 (0.74)	0.31 (0.34)	0.76 (0.46)
*Christensenellaceae R7 group*	0.39 (0.56)	0.49 (0.46)	0.38 (0.54)	0.47 (0.35)	1.15 (1.56)	0.6 (0.4)
*UCG010*	0.39 (0.43)	0.44 (0.6)	0.39 (0.36)	0.52 (0.85)	0.57 (0.29)	0.59 (0.49)
*Akkermansia*	0.38 (0.88)	0.4 (1.28)	0.52 (1.07)	0.86 (1.75)	0.23 (0.29)	0.15 (0.19)
*Streptococcus*	0.35 (0.46)	0.28 (0.58)	0.28 (0.6)	0.4 (0.57)	0.24 (0.11)	1.56 (1.72)
*Desulfovibrio*	0.35 (0.51)	0.36 (1.43)	0.21 (0.29)	0.27 (0.55)	0.08 (0.09)	0.01 (0.01)
*Catenibacterium*	0.3 (0.38)	0.3 (0.34)	0.3 (0.44)	0.25 (0.33)	0.59 (0.83)	0.98 (1.6)
*Butyrivibrio*	0.3 (0.71)	0.31 (0.65)	0.38 (0.87)	0.38 (0.65)	0.03 (0.05)	0.3 (0.46)
*Sutterella*	0.3 (0.37)	0.29 (0.25)	0.3 (0.33)	0.31 (0.37)	0.35 (0.44)	0.39 (0.18)
*Clostridium sensustricto1*	0.29 (0.74)	0.31 (0.72)	0.08 (0.13)	0.19 (0.35)	1.15 (0.74)	0.37 (0.7)
*Odoribacter*	0.25 (0.23)	0.26 (0.49)	0.22 (0.38)	0.3 (0.87)	0.36 (0.39)	0.1 (0.14)
*Lachnospiraceae UCG004*	0.22 (0.29)	0.21 (0.33)	0.24 (0.27)	0.21 (0.3)	0.2 (0.32)	0.41 (0.33)
*Eubacterium eligens group*	0.21 (0.38)	0.22 (0.57)	0.13 (0.15)	0.12 (0.2)	0.12 (0.09)	0.45 (0.7)
*Monoglobus*	0.21 (0.38)	0.3 (0.56)	0.53 (1.1)	0.31 (0.46)	0.43 (0.61)	0.2 (0.18)
*Megasphaera*	0.21 (0.54)	0.14 (0.34)	0.21 (0.64)	0.52 (1.16)	0.09 (0.18)	0.1 (0.15)
*Eubacterium ruminantium group*	0.21 (0.37)	0.17 (0.24)	0.24 (0.41)	0.4 (0.88)	0.12 (0.07)	0.15 (0.14)
*Bifidobacterium*	0.21 (0.46)	0.24 (0.44)	0.09 (0.12)	0.19 (0.25)	0.92 (1.91)	0.23 (0.25)
*Butyricimonas*	0.21 (0.3)	0.26 (0.3)	0.37 (0.77)	0.16 (0.16)	0.36 (0.27)	0.44 (0.24)
*Collinsella*	0.21 (0.55)	0.29 (0.75)	0.37 (0.98)	0.17 (0.34)	0.77 (1.56)	0.45 (0.91)
*Lachnospiraceae UCG001*	0.17 (0.37)	0.2 (0.26)	0.05 (0.08)	0.15 (0.19)	0.47 (0.84)	0.15 (0.19)
*Eubacterium xylanophilum group*	0.16 (0.65)	0.18 (0.31)	0.22 (0.52)	0.27 (0.35)	0.06 (0.08)	0.19 (0.4)
*Holdemanella*	0.16 (0.23)	0.24 (0.68)	0.15 (0.22)	0.11 (0.36)	0.44 (0.75)	0.15 (0.21)
*RF39*	0.15 (0.2)	0.17 (0.36)	0.13 (0.2)	0.18 (0.3)	0.14 (0.12)	0.4 (0.36)
*Acidaminococcus*	0.15 (0.28)	0.13 (0.21)	0.05 (0.09)	0.27 (0.64)	0.16 (0.17)	0.5 (0.64)
*Lachnoclostridium*	0.15 (0.34)	0.2 (0.26)	0.2 (0.34)	0.2 (0.32)	0.18 (0.16)	0.16 (0.15)
*Citrobacter*	0.14 (0.37)	0.15 (0.26)	0.03 (0.06)	0.13 (0.23)	0.02 (0.05)	0.15 (0.12)
*Allisonella*	0.13 (0.22)	0.16 (0.21)	0.06 (0.06)	0.12 (0.15)	0.08 (0.09)	0.17 (0.1)
*Parasutterella*	0.13 (0.52)	0.11 (0.17)	0.07 (0.18)	0.23 (0.59)	0.01 (0.02)	0.04 (0.04)
*Prevotellaceae NK3B31 group*	0.12 (0.24)	0.05 (0.44)	0.03 (0.07)	0.3 (0.93)	0 (0)	0 (0)
*Anaerostipes*	0.11 (0.17)	0.17 (0.61)	0.07 (0.12)	0.05 (0.16)	0.22 (0.2)	0.06 (0.14)
*Romboutsia*	0.11 (0.13)	0 (0.01)	0.16 (0.34)	0.81 (3.58)	0.14 (0.09)	0 (0.01)
*Lachnospira*	0.1 (0.16)	0.11 (0.12)	0.1 (0.17)	0.18 (0.21)	0.09 (0.05)	0.09 (0.05)
*Comamonas*	0.09 (0.17)	0.16 (0.29)	0.23 (0.84)	0.19 (0.39)	0.05 (0.07)	0.05 (0.04)
*Erysipelotrichaceae UCG003*	0.09 (0.23)	0.12 (0.54)	0.04 (0.07)	0.02 (0.04)	0.34 (0.67)	0.02 (0.02)
*Klebsiella*	0.08 (0.56)	0.07 (0.16)	0.06 (0.16)	0.08 (0.1)	0 (0)	0.16 (0.17)
*Eubacterium ventriosum group*	0.08 (0.12)	0.01 (0.05)	0.09 (0.17)	0.39 (1.56)	0.14 (0.21)	0.03 (0.06)
*Oscillibacter*	0.08 (0.10)	0.06 (0.12)	0.05 (0.07)	0.05 (0.09)	0.03 (0.03)	0.44 (0.42)
*Slackia*	0.07 (0.21)	0.08 (0.23)	0.02 (0.03)	0.09 (0.17)	0.28 (0.39)	0.05 (0.01)
*Weissella*	0.05 (0.16)	0.09 (0.42)	0.06 (0.17)	0 (0.01)	0.02 (0.03)	0.04 (0.1)
*Bilophila*	0.05 (0.21)	0.12 (0.38)	0.1 (0.21)	0.03 (0.08)	0.58 (1.16)	0.02 (0.04)
*Clostridiavadin BB60 group*	0.05 (0.11)	0.03 (0.14)	0.06 (0.09)	0.15 (0.4)	0.02 (0.02)	0.13 (0.21)
*Mitsuokella*	0.05 (0.09)	0.08 (0.16)	0.08 (0.15)	0.04 (0.08)	0.08 (0.11)	0.1 (0.1)
*Lachnospiraceae UCG003*	0.04 (0.14)	0.02 (0.08)	0.01 (0.02)	0.12 (0.53)	0 (0)	0 (0)
*Family XIIIUCG001*	0.04 (0.08)	0.04 (0.15)	0.03 (0.07)	0.02 (0.03)	0.04 (0.05)	0.26 (0.57)
*Ruminococcus gnavus group*	0.03 (0.11)	0.05 (0.11)	0.08 (0.14)	0.09 (0.3)	0 (0.01)	0.03 (0.04)
*Prevotellaceae UCG003*	0.03 (0.14)	0.07 (0.23)	0.11 (0.38)	0.03 (0.07)	0 (0)	0.04 (0.05)
*Fusobacterium*	0.02 (0.1)	0.03 (0.06)	0.06 (0.23)	0.04 (0.05)	0 (0.01)	0.04 (0.08)
*Anaerovibrio*	0.01 (0.06)	0.01 (0.04)	0 (0.01)	0.07 (0.13)	0 (0)	0 (0)
*Lactobacillus*	0.01 (0.06)	0.02 (0.08)	0 (0)	0.05 (0.23)	0.1 (0.13)	0 (0)
*Hungatella*	0.01 (0.04)	0.03 (0.18)	0.01 (0.05)	0 (0.01)	0 (0)	0 (0)
*Tyzzerella*	0.01 (0.02)	0.02 (0.05)	0.01 (0.02)	0.01 (0.02)	0 (0)	0.01 (0.01)
*Raoultella*	0.01 (0.03)	0.03 (0.16)	0 (0)	0 (0)	0.07 (0.15)	0 (0)
*Megamonas*	0 (0.02)	0.01 (0.04)	0 (0.01)	0.01 (0.03)	0 (0)	0.09 (0.21)
*Treponema*	0 (0)	0 (0)	0 (0.02)	0.05 (0.24)	0.01 (0.02)	0 (0)
*Asteroleplasma*	0 (0)	0.01 (0.02)	0 (0)	0.02 (0.05)	0 (0)	0 (0.01)
*Paeniclostridium*	0 (0)	0 (0.03)	0 (0)	0 (0)	0 (0)	0 (0.01)

Data are mean (±SD). *p*-values are based on the Wilcoxon signed-rank test for paired samples.
